# DNA Repair Pathways and Their Association With Lethal Prostate Cancer in African American and European American Men

**DOI:** 10.1093/jncics/pkab097

**Published:** 2021-12-27

**Authors:** Anna Plym, Miklós Dióssy, Zoltan Szallasi, Oliver Sartor, Jonathan Silberstein, Isaac J Powell, Timothy R Rebbeck, Kathryn L Penney, Lorelei A Mucci, Mark M Pomerantz, Adam S Kibel

**Affiliations:** Urology Division, Department of Surgery, Brigham and Women’s Hospital, Harvard Medical School, Boston, MA, USA; Department of Epidemiology, Harvard T.H. Chan School of Public Health, Boston, MA, USA; Department of Medical Epidemiology and Biostatistics, Karolinska Institutet, Stockholm, Sweden; Translational Cancer Genomics, Danish Cancer Society Research Center, Copenhagen, Denmark; Translational Cancer Genomics, Danish Cancer Society Research Center, Copenhagen, Denmark; Computational Health Informatics Program, Boston Children’s Hospital, Boston, MA, USA; 2nd Department of Pathology, SE NAP, Brain Metastasis Research Group, Semmelweis University, Budapest, Hungary; Department of Medicine, Tulane Cancer Center, Tulane University School of Medicine, New Orleans, LA, USA; Section of Urology and Uro-Oncology, Memorial Healthcare System, Broward, FL, USA; Department of Urology, Wayne State University, Detroit, MI, USA; Department of Epidemiology, Harvard T.H. Chan School of Public Health, Boston, MA, USA; Department of Medical Oncology, Dana Farber Cancer Institute, Boston, MA, USA; Department of Epidemiology, Harvard T.H. Chan School of Public Health, Boston, MA, USA; Channing Division of Network Medicine, Department of Medicine, Brigham and Women’s Hospital, Harvard Medical School, Boston, MA, USA; Department of Epidemiology, Harvard T.H. Chan School of Public Health, Boston, MA, USA; Department of Medical Oncology, Dana Farber Cancer Institute, Boston, MA, USA; Urology Division, Department of Surgery, Brigham and Women’s Hospital, Harvard Medical School, Boston, MA, USA

## Abstract

**Background:**

Altered DNA damage response (DDR) has emerged as an important mechanism for the development of aggressive prostate cancer among men of European ancestry but not other ancestry groups. Because common mechanisms for aggressive disease are expected, we explored a large panel of DDR genes and pathways to demonstrate that DDR alterations contribute to development of aggressive prostate cancer in both African American and European American men.

**Methods:**

We performed a case-case study of 764 African American and European American men with lethal or indolent prostate cancer treated at 4 US hospitals. We calculated carrier frequencies of germline pathogenic or likely pathogenic sequence variants within 306 DDR genes, summarized by DDR pathway, and compared lethal cases against indolent cases using 2-sided Fisher’s exact tests. Secondary analysis examined if carrier frequencies differed by ancestry.

**Results:**

Lethal cases were more likely to carry a pathogenic sequence variant in a DDR gene compared with indolent cases (18.5% vs 9.6%, *P* = 4.30 × 10^−4^), even after excluding *BRCA2* (14.6% vs 9.6%, *P* = .04). The carrier frequency was similar among lethal cases of African (16.7% including and 15.8% excluding *BRCA2*) and lethal cases of European (19.3% including and 14.2% excluding *BRCA2*) ancestry. Three DDR pathways were statistically significantly associated with lethal disease: homologous recombination (*P* = .003), Fanconi anemia (*P* = .002), and checkpoint factor (*P* = .02).

**Conclusions:**

Our findings suggest that altered DDR is an important mechanism for aggressive prostate cancer not only in men of European but also of African ancestry. Therefore, interrogation of entire DDR pathways is needed to fully characterize and better define genetic risk of lethal disease.

Altered DNA damage response (DDR) has emerged as an important mechanism for the development of aggressive prostate cancer. Several studies have reported enrichment of rare germline pathogenic variants within DDR genes, particularly *BRCA2*, among men with advanced prostate cancer (1-5). Pritchard et al. (3) examined a panel of 20 DDR genes in men predominately of European ancestry and demonstrated that 11.8% of men with metastatic prostate cancer carried a DDR gene alteration, whereas the corresponding frequency was only 4.6% among men with localized prostate cancer and 2.7% among men without a diagnosis of prostate cancer. In the largest sequencing study of men with prostate cancer performed to date, including 5545 men of European ancestry, of which 467 men had metastatic disease, Darst et al. (2) showed that 14.2% of aggressive cases and 15.4% of metastatic cases carried a pathogenic variant within 24 analyzed DDR genes, in contrast to 10.6% of nonaggressive comparison cases. The individual genes most strongly associated with aggressive disease in men of European ancestry are *BRCA2*, *PALB2, ATM*, and *CHEK2* (2,4,6).

Less is known regarding the role of DDR genes in the development of aggressive prostate cancer among men of other ancestry groups. This is particularly important in men of African ancestry. African American men have higher prostate cancer incidence and mortality rates than European American men (7), and genetic factors likely play a role. In the only larger sequencing study performed in men of African ancestry, including 2098 men with prostate cancer—of which 73 men had documented metastatic disease—Matejcic et al. (8) observed that 5.7% of men with metastatic prostate cancer were carriers of a pathogenic variant in a panel of 19 DDR genes. The frequency was lower in men with low- or intermediate-risk prostate cancer (2.3%) and in men without prostate cancer (2.1%). Although these findings indicate that DDR genes may be of importance also for men of African ancestry, further studies including a larger number of metastatic cases and a more extensive gene panel are needed to better understand the role of DDR genes in men of African ancestry and which genes and pathways are most frequently altered.

With the ultimate goal of identifying genetic markers that can predict disease course and personalize treatment for men of all ancestries, we sought to identify the DDR pathways associated with lethal prostate cancer in African American and European American men. We hypothesized that underlying mechanisms for aggressive disease are similar and that DDR gene alterations in general contribute to development of aggressive prostate cancer irrespective of ancestry.

## Methods

### Study Patients

We conducted an unmatched case-case study of African American and European American men with lethal or indolent prostate cancer who had previously provided a blood or buccal swab tissue sample and had been treated at one of the following hospitals up until 2016: Dana-Farber Cancer Institute and Brigham and Women’s Hospital, the Hospital of the University of Pennsylvania, Tulane University, and Wayne State University. Information on self-reported race and ethnicity was extracted from medical records and subsequently verified using ancestral markers as described below. Lethal cases included men diagnosed with metastatic disease (at diagnosis or during follow-up) or who had died from prostate cancer. Indolent cases were selected based on their clinical characteristics and included men treated with curative intent or undergoing active surveillance, with Gleason score 6 tumors, and without evidence of recurrence or additional treatment at 5 years. Of the 837 men originally included in the sample, 24 men who did not fulfil the inclusion criteria for lethal disease (n = 1) or indolent cancer (n = 23) were removed following a medical chart review. Fourteen control men who had biopsy Gleason 6 cancer but were upgraded at radical prostatectomy to Gleason 3 with a small quantity of Gleason 4 were included. Of the 813 men remaining, 764 (94.0%) were included for analysis after successful sequencing and quality control as outlined below.

All patients provided written informed consent, and the project was approved by the institutional review board committees at each site. The project as a whole was approved by the Brigham and Women’s Hospital Institutional Review Board (2015P002118).

### Sequencing

Targeted sequencing of 306 preselected DDR genes was done at the McDonnell Genome Institute at Washington University in St. Louis (details in the Supplementary Methods, available online). One blood sample and 31 buccal swab samples failed the initial quality control.

### Alignment, Postprocessing, and Variant Calling

Samples were aligned to grch38 using bwa-mem (v0.7.15) (9) and postprocessed according to the genome analysis toolkit (GATK, v.4.1.0.0) (10) best practices workflow. Duplicates were flagged using sambamba-markdup (v.0.6.7) (11), and base quality recalibration was executed using GATK BaseRecalibrator. The postprocessed mean sequencing depth at the targeted regions was 187.8×, the median depth was 155×. Variants were called using GATK HaplotypeCaller with additional hard filters that required the depth at the variant sites to be at least 15× and the base quality scores of the supporting reads to be at least 20. Pathogenic and likely pathogenic variants (PSVs) were identified following the guidelines of the American College of Medical Genetics and Genomics and the Association for Molecular Pathology by using Intervar (12). Five buccal swab samples were identified as outliers in terms of number of genetic alterations detected in them and hence were removed from the study.

### Confirmation of Ancestry Groups

To ensure that the self-reported information on race and ethnicity corresponded to either African or European ancestry groups, 210 single-nucleotide polymorphisms were collected from the ExAC database (13) that were abundant in either ancestry group but mostly absent in the other (N_EU_ = 102, N_AF_ = 108), and their loci overlapped with the targeted regions. The genotypes of these locations were collected into a single matrix, and the principal components (PCs) of the dataset were identified using a singular value decomposition approach. We found that the first 2 eigenvectors separated the 2 ancestries almost perfectly. Outliers were identified using a k-nearest neighbors (k = 15) approach, measured in the PC1-PC2 plane, and were subsequently removed (n = 12).

### DDR Genes and Pathways

Based on literature and database searches (14,15), genes were assigned to the following traditional core DDR pathways: base excision repair, nucleotide excision repair, mismatch repair (MMR), Fanconi anemia (FA), homologous recombination (HR), nonhomologous end joining, direct repair, and translesion synthesis (Table 2). We also included noncore DDR pathways (14), which involved the checkpoint factor pathway, other noncore pathways, and a separate group consisting of genes classified as probable DDR associated. Because genes are often important in multiple pathways, genes were represented in more than one.

**Table 1.** Demographic and clinical characteristics of the 764 men included for analysis

Characteristic	Lethal cases (n = 410)	Indolent cases (n = 354)
Ancestry group, No. (%)		
African American	114 (27.8)	74 (20.9)
European American	296 (72.2)	280 (79.1)
DNA from blood, No. (%)	405 (98.8)	345 (97.5)
Hospital, No. (%)		
Dana-Farber/Brigham and Women’s Hospital	348 (84.9)	306 (86.4)
Hospital of the University of Pennsylvania	5 (1.2)	9 (2.5)
Tulane University	18 (4.4)	0 (0.0)
Wayne State University	39 (9.5)	39 (11.0)
Median age at diagnosis (IQR)^a^, y	62.7 (57.0, 68.2)	58.2 (53.5, 63.5)
Median age at lethal event (IQR)^b^, y	66.9 (59.4, 73.6)	—^d^
M stage at diagnosis, No. (%)		
M0	212 (51.7)	354 (100.0)
M1	150 (36.6)	0 (0.0)
Unknown	48 (11.7)	0 (0.0)
T stage at diagnosis, No. (%)		
T1	214 (52.2)	241 (68.1)
T2	49 (12.0)	47 (13.3)
T3	17 (4.1)	7 (2.0)
T4	7 (1.7)	0 (0.0)
Unknown	123 (30.0)	59 (16.7)
Median PSA at diagnosis (IQR)^c^, ng/mL	13.5 (6.2, 56.8)	4.6 (3.6, 5.7)
Final Gleason Grade Group, No. (%)		
1 (Gleason score ≤6)	31 (7.6)	340 (96.0)
2 (Gleason score 3 + 4)	58 (14.1)	14 (4.0)
3 (Gleason score 4 + 3)	43 (10.5)	0 (0.0)
4 (Gleason score 8)	70 (17.1)	0 (0.0)
5 (Gleason score 9-10)	149 (36.3)	0 (0.0)
Unknown	59 (14.4)	0 (0.0)
Primary treatment, No. (%)		
Radical prostatectomy	116 (28.3)	335 (94.6)
External beam radiation only	29 (7.1)	8 (2.3)
External beam radiation with hormones	55 (13.4)	0 (0.0)
Brachytherapy	10 (2.4)	2 (0.6)
Active surveillance	2 (0.5)	9 (2.5)
Hormones alone	132 (32.2)	0 (0.0)
Other	49 (12.0)	0 (0.0)
Unknown	17 (4.1)	0 (0.0)

a Age at diagnosis was missing for 2.9% of lethal cases and 3.4% of indolent cases. IQR = interquartile range; PSA = prostate-specific antigen.

b Age at lethal disease was missing for 9.3% of lethal cases.

c PSA at diagnosis was missing for 16.3% of lethal cases and 8.8% of indolent cases.

d Not applicable.

**Table 2.** DNA damage response genes with pathogenic sequence variants in men with lethal and indolent prostate cancer^a^

Core DDR pathways								Noncore DDR pathways		
HR	FA	NHEJ	MMR	NER	BER	TLS	DR	Check-point factor	Other noncore	Probable associated
*BLM*	*BARD1*	*APTX*	*EXO1*	*DDB2*	*APTX*	*POLH*	*ASCC1*	*ATM*	*BLM*	*CCNO*
*BRCA2*	*BLM*	*ATM*	*MLH1*	*ERCC2*	*MUTYH*			*ATR*	*MRE11*	*POLG*
*MRE11*	*BRCA2*	*DCLRE1C*	*MLH3*	*ERCC3*	*NTHL1*			*PER2*	*NBN*	*VCP*
*NBN*	*BRIP1*	*MRE11*	*MSH2*	*ERCC5*	*PNKP*			*RAD50*	*RAD50*	
*RAD50*	*FANCA*	*NBN*	*MSH6*	*ERCC8*	*POLE*			*RBBP8*	*TP53*	
*RAD51*	*FANCI*	*PNKP*	*PMS2*	*POLE*	*POLH*					
*RAD51C*	*RAD51*	*RAD50*		*XPA*	*UNG*					
*RAD54B*	*RAD51C*	*XRCC1*		*XRCC1*	*XRCC1*					
*RAD54L*		*XRCC4*								
*RECQL4*										
*SLX4*										
*UIMC1*										

a BER = base excision repair; DDR = DNA damage response; DR = direct repair; FA = Fanconi anemia; HR = homologous recombination; MMR = mismatch repair; NER = nucleotide excision repair; NHEJ = nonhomologous end joining; TLS = translesion synthesis.

### Statistical Analysis

Carrier frequencies of PSVs were calculated by gene and summarized by DDR pathway. Lethal cases were compared against indolent cases using 2-sided Fisher’s exact tests, with *P* less than .05 considered as statistically significant. Given the sample size and rarity of variants, hypothesis testing and *P* value calculation were limited to pathway-based analysis only. In secondary analyses, comparisons were made stratified by ancestry and age at diagnosis. We further used logistic regression to calculate odds ratios (ORs) and 95% confidence intervals (CIs) for the association between carrying any PSV within a DDR gene and lethal prostate cancer, adjusting for age at diagnosis and PCs 1-3. A model including an interaction term with ancestry was fitted to test for effect modification by ancestry. Variance of unknown clinical significance (VUS) was examined in a sensitivity analysis that included the most likely pathogenic variants among the VUS (details in the Supplementary Methods, available online).

## Results

Of the 764 prostate cancer cases included for analysis, 188 (24.6%) were African American and 576 (75.4%) European American. Among the 410 lethal cases, the median age at diagnosis was 62.7 years and the median age at lethal event (metastatic disease or prostate cancer–specific death) was 66.9 years (Table 1). The most common primary treatment was hormones alone (32.2%) followed by radical prostatectomy (28.3%). Likely reflecting the better outcome, the 354 indolent cases were diagnosed at a younger age than the lethal cases, at a median age of 58.2 years. The vast majority of indolent cases (94.6%) had been treated with radical prostatectomy.

Within the 306 sequenced DDR genes, 95 unique PSVs were identified (Supplementary Table 1, available online). The variants were distributed within 47 DDR genes (Supplementary Table 2, available online) and 11 DDR pathways (Table 2). Most men (97.4%) carrying a PSV had only 1 alteration.

**Figure 1.**
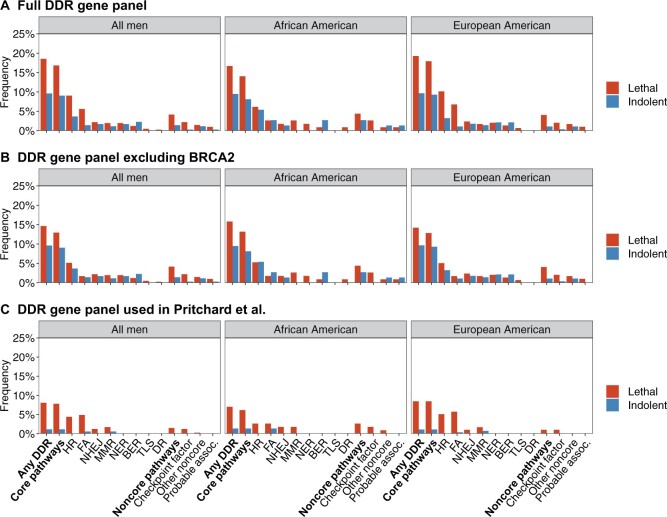
Carrier frequencies of pathogenic sequence variants in lethal and indolent cases summarized by DNA damage response (DDR) pathway. **A**) Full DDR gene panel. **B**) DDR gene panel excluding *BRCA2*. **C**) Gene panel from Pritchard et al (3). BER = base excision repair; DR = direct repair; FA = Fanconi anemia; HR = homologous recombination; MMR = mismatch repair; NER = nucleotide excision repair; NHEJ = nonhomologous end joining; TLS = translesion synthesis.

**Figure 2.**
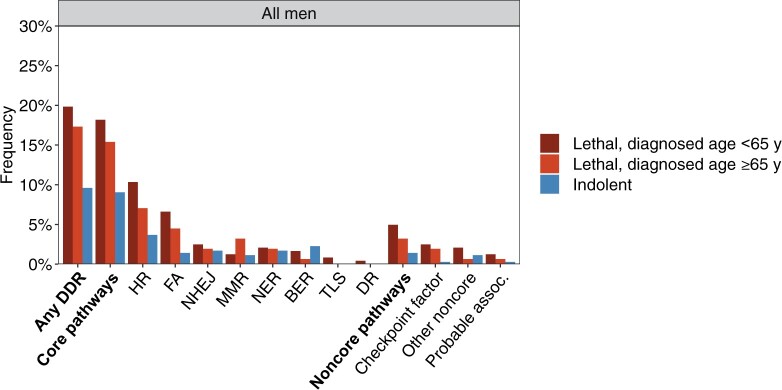
Carrier frequencies of pathogenic sequence variants in lethal cases diagnosed younger than 65 years, lethal cases diagnosed at 65 years and older, and indolent cases summarized by DNA damage response (DDR) pathway. BER = base excision repair; DR = direct repair; FA = Fanconi anemia; HR = homologous recombination; MMR = mismatch repair; NER = nucleotide excision repair; NHEJ = nonhomologous end joining; TLS = translesion synthesis.

Overall, 18.5% of lethal cases and 9.6% of indolent cases carried at least 1 PSV in a DDR gene (*P* = 4.30 × 10^−4^) (Figure 1, A;  Table 3). The carrier frequency was not statistically significantly different among lethal cases of African ancestry (16.7%) than among lethal cases of European ancestry (19.3%) (*P*_interaction_ = .73). In adjusted analyses, the odds ratio was 2.30 (95% CI = 1.44 to 3.66) for the combined group, 1.81 (95% CI = 0.67 to 4.86) for the African ancestry group, and 2.49 (95% CI = 1.47 to 4.22) for the European ancestry group. Analyses stratified by age at diagnosis demonstrated an even higher carrier frequency in lethal cases diagnosed before age 65 years (19.8%) (Figure 2). This pattern was not observed in men of African ancestry, although the smaller sample size may limit the interpretation of this finding (Supplementary Table 3, available online). An additional examination of age at lethal event instead of age at diagnosis showed a similar pattern (Supplementary Table 4, available online).

**Table 3.** Carrier frequencies of pathogenic sequence variants in lethal and indolent cases summarized by DNA damage response pathway

Pathway	All men			African American			European American		
	Lethal cases, No. (%)	Indolent cases, No. (%)	*P* ^a^	Lethal cases, No. (%)	Indolent cases, No. (%)	*P* ^a^	Lethal cases, No. (%)	Indolent cases, No. (%)	*P* ^a^
Any DDR pathway	76 (18.5)	34 (9.6)	4.30 × 10^–4^	19 (16.7)	7 (9.5)	.20	57 (19.3)	27 (9.6)	.001
Core pathways	69 (16.8)	32 (9.0)	.002	16 (14.0)	6 (8.1)	.25	53 (17.9)	26 (9.3)	.003
HR	37 (9.0)	13 (3.7)	.003	7 (6.1)	4 (5.4)	1.00	30 (10.1)	9 (3.2)	.001
FA	23 (5.6)	5 (1.4)	.002	3 (2.6)	2 (2.7)	1.00	20 (6.8)	3 (1.1)	4.20 × 10^–04^
NHEJ	9 (2.2)	6 (1.7)	.80	2 (1.8)	1 (1.4)	1.00	7 (2.4)	5 (1.8)	.77
MMR	8 (2.0)	4 (1.1)	.40	3 (2.6)	0 (0.0)	.28	5 (1.7)	4 (1.4)	1.00
NER	8 (2.0)	6 (1.7)	1.00	2 (1.8)	0 (0.0)	.52	6 (2.0)	6 (2.1)	1.00
BER	5 (1.2)	8 (2.3)	.40	1 (0.9)	2 (2.7)	.56	4 (1.4)	6 (2.1)	.54
TLS	2 (0.5)	0 (0.0)	.50	0 (0.0)	0 (0.0)	—^b^	2 (0.7)	0 (0.0)	.50
DR	1 (0.2)	0 (0.0)	1.00	1 (0.9)	0 (0.0)	1.00	0 (0.0)	0 (0.0)	—^b^
Noncore pathways	17 (4.1)	5 (1.4)	.03	5 (4.4)	2 (2.7)	.71	12 (4.1)	3 (1.1)	.03
Checkpoint factor	9 (2.2)	1 (0.3)	.02	3 (2.6)	0 (0.0)	.28	6 (2.0)	1 (0.4)	.12
Other noncore	6 (1.5)	4 (1.1)	.76	1 (0.9)	1 (1.4)	1.00	5 (1.7)	3 (1.1)	.73
Probable associated	4 (1.0)	1 (0.3)	.38	1 (0.9)	1 (1.4)	1.00	3 (1.0)	0 (0.0)	.25

a *P* values from 2-sided Fisher’s exact test comparing lethal cases with indolent cases. BER = base excision repair; DDR = DNA damage response; DR = direct repair; FA = Fanconi anemia; HR = homologous recombination; MMR = mismatch repair; NER = nucleotide excision repair; NHEJ = nonhomologous end joining; TLS = translesion synthesis.

b Not applicable.

*BRCA2* is well known to be associated with aggressive prostate cancer. To determine if additional DDR alterations were associated with risk, we repeated the analysis excluding PSVs in *BRCA2*. The carrier frequency of having at least 1 PSV in a DDR gene was 14.6% in lethal cases overall compared with 9.6% in indolent cases (*P* = .04) (Figure 1, B;  Supplementary Table 5, available online), demonstrating that the increased frequency of DDR alterations among lethal cases is not solely due to *BRCA2* alterations. Pathogenic *BRCA2* alterations were less frequent among lethal cases of African ancestry (0.9%) compared with lethal cases of European ancestry (5.1%) (Supplementary Table 2, available online). Excluding PSVs in *BRCA2*, the carrier frequency of DDR gene alterations was similar among lethal cases of African (15.8%) and European (14.2%) ancestry. Using the gene panel from Pritchard et al. (3), the carrier frequency among lethal cases was 8.0% (combined group), 7.0% (African), and 8.4% (European) (Figure 1, C;  Supplementary Table 6, available online).

Three pathways were statistically significantly associated with lethal disease in the combined group of men: HR, FA, and checkpoint factor (Figure 1, A;  Table 3). Whereas lethal cases of either ancestry presented with an enrichment of PSVs within genes in the checkpoint factor pathway, the associations between lethal disease and HR and FA were primarily driven by strong associations among men of European ancestry. However, after removing *RAD54L*, which was the most frequently altered DDR gene in indolent cases, HR appeared to be of importance also for men of African ancestry with a carrier frequency of 6.1% among lethal cases and 2.7% among indolent cases (Supplementary Table 7, available online). Among men of African ancestry, only lethal cases had PSVs within genes belonging to the checkpoint factor and the MMR pathway.

**Figure 3.**
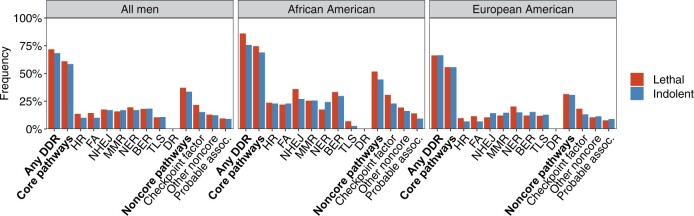
Carrier frequencies of the most likely pathogenic variants among the variants of unknown clinical significance in lethal and indolent cases summarized by DNA damage response (DDR) pathway. BER = base excision repair; DR = direct repair; FA = Fanconi anemia; HR = homologous recombination; MMR = mismatch repair; NER = nucleotide excision repair; NHEJ = nonhomologous end joining; TLS = translesion synthesis.

In the sensitivity analysis of VUS, lethal cases had a marginally higher carrier frequency (71.7%) compared with indolent cases (68.4%, *P* = .34), with the highest carrier frequency among lethal African American cases (Figure 3; Supplementary Table 8, available online). Similar to the main analysis, lethal cases had a statistically significantly higher carrier frequency of VUS within the checkpoint factor pathway (*P* = .03), suggesting that not all pathogenic sequence variants are captured by current classification. In addition to VUS within the checkpoint factor pathway, we observed a possible enrichment of VUS among lethal cases within *BRCA1*, *SETX*, and *DCLRE1C* (Supplementary Table 9, available online).

## Discussion

We performed the most comprehensive analysis to date on the role of inherited DDR genes in aggressive prostate cancer—including a panel of over 300 DDR genes—and demonstrated high carrier frequency of DDR gene alterations in lethal cases irrespective of ancestry. Except for *BRCA2*, there was no evidence of DDR genes being less relevant for African American men than for European American men, consistent with the expectation that altered DDR is an important mechanism in the development of aggressive prostate cancer in all ancestry groups. Three DDR pathways were statistically significantly associated with lethal disease in the combined group of men: HR, FA, and checkpoint factor.

To the best of our knowledge, our study is the first study examining germline alterations within a large set of DDR genes in both African American and European American men with lethal prostate cancer. Only a few previous studies have included lethal events among men of African ancestry, all with considerably smaller panels of 3 to 19 DDR genes and without a comparison with European cases and/or nonaggressive cases (8,16,17). Although smaller gene panels (including the panel used by Pritchard et al.) (3) appear to capture the excess risk of lethal disease associated with DDR genes among men with African ancestry, our study also points to *BRCA2* gene alterations being less relevant and that additional genes and DDR pathways are likely involved. In particular, the checkpoint factor pathway (which includes *ATM*, *ATR*, and *PER2*) and the MMR pathway (which includes *MLH3* and *MSH2*) may be of special interest for men of African ancestry given the observation of PSVs among lethal cases only, for whom carrier frequencies of 2.6% were observed. *ATM* and the MMR pathway have previously been pointed out as potentially relevant for prostate cancer etiology and aggressiveness among men of African ancestry (8,18). *ATR* and *PER2,* a circadian clock gene that help regulate DDR (19), have previously been associated with metastatic and lethal disease in men of European ancestry (6,20).

Consistent with Darst et al. (2), who had a larger sample size overall but a similar number of metastatic cases as our study, we identified the HR and FA pathways as important for the development of lethal prostate cancer in men of European ancestry. *BRCA2* is involved in both of these pathways and appears to be a main driver of this association (2). Expanding on the work of Darst et al. (2), our study highlights a third pathway of importance, the checkpoint factor pathway. Within this pathway, germline alterations in *ATM* were most frequent, consistent with previous studies conducted in European populations (2-4,21). In addition to *BRCA2* and *ATM*, we observed a possible enrichment of PSVs within *CCNO* among lethal cases of European ancestry. CCNO (Cyclin O) is involved in cell cycle regulation and has been associated with DNA damage-induced apoptosis (22). Its role in prostate cancer is largely unknown, but there are reports of elevated expression for other cancers (23). *BRCA2*, *ATM*, and *CCNO* alterations were all enriched in lethal cases diagnosed before the age of 65 years, contributing to the high carrier frequency of DDR gene alterations among lethal cases within this age group. Although DDR gene alterations were most frequent among lethal prostate cancer cases, our study and others (2,3,8) point to an enrichment also among nonaggressive prostate cancer cases, supporting a general role of DDR in prostate cancer etiology.

Our study does not support the hypothesis that differences in DDR gene alterations are the main drivers of the observed disparities in survival between men of European and African ancestry. The frequency of DDR gene alterations was similar across the 2 ancestry groups, among both lethal (15.8% for African and 14.2% for European lethal cases after excluding *BRCA2*) and indolent cases (9.5% for African and 9.6% for European indolent cases). However, our analysis is based on a variant classification of genes that relies on data from European ancestry groups, and it is possible that VUS matters for African ancestry groups. Data from both our study and others (24) indicate higher proportions of VUS among African ancestry groups, and a future reclassification may reveal a different pattern. In addition, DDR genes cannot explain the full picture of aggressive prostate cancer; other inherited factors may be involved, including common variants reported to be more frequent in African ancestry groups than in other ancestry groups (25-28).

Our study has some limitations. Although our study suggests that DDR genes are important for both African American and European American men, with no difference in the prevalence of DDR gene alterations, the small sample size for African American men limits the ability to draw strong conclusions supported by statistical evidence for this ancestry group. As previously exemplified, studies of rare genetic variants would ideally require sample sizes in the order of 25* *000 men to have sufficient statistical power (2), which will be difficult to acquire. Another potential limitation was our definition of control men. Although few men undergoing radical prostatectomy with Gleason 6 disease die of prostate cancer, it is possible that some might have developed lethal disease if untreated. Also, men with indolent cancer were diagnosed and treated at a younger age than men with lethal disease, potentially underestimating the association between DDR and lethal prostate cancer. We further lacked clinical data at diagnosis, including stage of disease, for a subset of lethal cases. However, we do not believe that this affects the interpretation of results—all lethal cases were either diagnosed with metastatic disease or developed metastatic disease post diagnosis. Despite these limitations, our study is the first study—to our knowledge—allowing for a comparison of a large number of DDR genes in men with lethal prostate cancer of African and European ancestry with all men sequenced and annotated using the same technology and the same set of genes.

Our study suggests that rare genetic variants in DDR genes are implicated in lethal prostate cancer in men of African and European ancestry. Diagnostic and therapeutic approaches targeting DDR are likely to be beneficial for both populations. Although the HR and FA pathways were most strongly as sociated with lethal disease among men of European ancestry, genes within the checkpoint factor pathway may be of importance for men of either ancestry. Further interrogation of DDR pathways important for the development of aggressive prostate cancer across ancestry groups can lead the way to better definitions of high-risk men and new approaches for prevention and treatment.

## Funding

This work was supported by the DiNovi Family Foundation, William Casey, and the Swedish Society for Medical Research (P19-0017).

## Notes

**Role of the funder:** The funder had no role in the design of the study; the collection, analysis, and interpretation of the data; the writing of the manuscript; and the decision to submit the manuscript for publication.

**Disclosures:** The authors report no conflict of interest.

**Author contributions:** AP: conceptualization, data curation, formal analysis, investigation, methodology, software, visualization, writing—original draft. MK: conceptualization, data curation, formal analysis, investigation, methodology, software, visualization, writing—review and editing. ZS: conceptualization, investigation, methodology, validation, writing—review and editing. OS: conceptualization, investigation, resources, validation, writing—review and editing. JS: conceptualization, investigation, resources, validation, writing—review and editing. IJP: conceptualization, investigation, resources, validation, writing—review and editing. TRR: conceptualization, investigation, resources, validation, writing—review and editing. KLP: conceptualization, investigation, validation, writing—review and editing. LAM: conceptualization, investigation, validation, writing—review and editing. MMP: conceptualization, investigation, resources, validation, writing—review and editing. ASK: conceptualization, funding acquisition, methodology, project administration, supervision, investigation, resources, validation, writing—review and editing.

**Prior presentations:** Preliminary result from this study has been presented at the Annual Dana-Farber/Harvard Cancer Center Celebration of Early Investigators in Cancer Research on January 21, 2021.

## Data Availability

The data underlying this article cannot be shared publicly due to the privacy of individuals that participated in the study. The data will be shared on reasonable request to the corresponding author.

## Supplementary Material

pkab097_Supplementary_Data
