# Supplementary material for: DNA Repair Pathways and Their Association With Lethal Prostate Cancer in African American and European American Men
Source: JNCI Cancer Spectr. 2021 Dec 27;6(1):pkab097. doi: 10.1093/jncics/pkab097 (PMC8784166; doi:10.1093/jncics/pkab097)
Supplement: pkab097_Supplementary_Data [file pkab097_supplementary_data.pdf]

Supplementary Material

Supplementary Methods.....2

Supplementary Table 1. All pathogenic and likely pathogenic sequence variants. 3

Supplementary Table 2. Carrier frequencies for pathogenic sequence variants by DNA damage response gene. 7

Supplementary Table 3. Association results by DNA damage response pathway and age at diagnosis.....9

Supplementary Table 4. Association results by DNA damage response pathway and age at lethal event. 10

Supplementary Table 5. Association results by DNA damage response pathway excluding *BRCA2*.....11

Supplementary Table 6. Association results by DNA damage response pathway including only genes by Pritchard et al. 12

Supplementary Table 7. Association results by DNA damage response pathway excluding *RAD54L*.....13

Supplementary Table 8. Association results for the most likely pathogenic variants among the variants of unknown clinical significance (VUS) by DNA damage response pathway.....14

Supplementary Table 9......15

Supplementary Methods

Genetic sequencing

100-250ng of genomic DNA was fragmented using the Covaris LE220 instrument targeting 250bp inserts. Automated dual indexed libraries were constructed utilizing the KAPA HTP Library Kit (Roche) on the SciClone NGS instrument (Perkin Elmer). Adaptor ligated fragments were PCR amplified with KAPA Hi Fi for 8-10 PCR cycles (250ng input-8 cycles; 100ng input-10 cycles). The size and mass of the libraries were assessed on the LabChip GX (Perkin Elmer). 85 libraries were pooled prior to hybridization, yielding a 5µg library pool. Ten library pools were hybridized with a custom set of Nimblegen probes, including coding regions and +/- 500bp from the transcription start site for the selected genes, which led to a 3.1Mb target space. The concentration of each captured library pool was accurately determined through qPCR according to the manufacturer's protocol (KAPA Biosystems) to produce cluster counts appropriate for the Illumina NovaSeq6000 platform. 2x150 paired end sequence data yielded ~1.8Gb of data per sample. Approximately 150x mean depth of coverage was achieved.

Variants of unknown significance

Among the variants of unknown clinical significance (VUS), we filtered the most likely pathogenic based on the following criteria: exon region, splicing region, non-coding RNA exon region, 3′UTR region, or 5′UTR region, and fulfilling at least three of these criteria: SIFT score $\leq$ 0.05, MetaSVM score $\geq$ -0.5, GERP RS score $<$ 0, and CADD phred $\geq$ 20.

Supplementary Table 1. All pathogenic and likely pathogenic sequence variants.

CHROM[a]	POS	REF	ALT	RS_ID	Ref. Gene	Function	Exonic function	African American		European American	
								Lethal cases, No.	Indolent cases, No.	Lethal cases, No.	Indolent cases, No.
1	45331223	C	A	– [b]	*MUTYH*	exonic	stopgain	1	0	0	0
1	46260761	G	A	rs145441107	*RAD54L*	exonic	nonsynonymous SNV	0	1	0	0
1	46260853	C	T	rs28363218	*RAD54L*	exonic	nonsynonymous SNV	0	1	3	2
1	46273746	A	G	– [b]	*RAD54L*	exonic	nonsynonymous SNV	0	0	0	1
1	46274182	G	A	rs746153043	*RAD54L*	exonic	nonsynonymous SNV	0	0	0	1
1	46277917	C	G	rs139712079	*RAD54L*	exonic	nonsynonymous SNV	0	0	1	0
1	241872074	C	A	– [b]	*EXO1*	exonic	stopgain	0	0	0	1
2	47403351	G	C	– [b]	*MSH2*	exonic	nonsynonymous SNV	0	0	1	0
2	47478448	C	A	– [b]	*MSH2*	exonic	nonsynonymous SNV	0	0	0	1
2	47478448	CT	C	rs63749983	*MSH2*	exonic	deletion	0	0	1	0
2	47478463	A	G	– [b]	*MSH2*	exonic	nonsynonymous SNV	1	0	0	0
2	47482858	C	G	rs267608022	*MSH2*	exonic	nonsynonymous SNV	1	0	0	0
2	47783243	C	T	rs786201042	*MSH6*	exonic	stopgain	0	0	1	0
2	127272934	CT	C	rs753182861	*ERCC3*	exonic	deletion	0	0	1	1
2	127280620	G	A	rs150954655	*ERCC3*	exonic	stopgain	1	0	0	0
2	127292756	G	A	rs34295337	*ERCC3*	exonic	stopgain	0	0	0	1
2	214745842	G	A	rs587780021	*BARD1*	exonic	stopgain	0	0	2	0
2	238277852	G	A	– [b]	*PER2*	exonic	stopgain	1	0	0	0
3	37047595	C	G	rs63750876	*MLH1*	exonic	nonsynonymous SNV	0	0	0	1
3	142459225	A	G	rs200556378	*ATR*	splicing	– [b]	1	0	0	0
5	55232413	GCAGC	G	rs759515939	*CCNO*	exonic	deletion	0	0	1	0
5	55233261	T	TGGGCC	rs587777502	*CCNO*	exonic	frameshift insertion	0	0	1	0

CHROM[a]	POS	REF	ALT	RS_ID	Ref. Gene	Function	Exonic function	African American		European American	
								Lethal cases, No.	Indolent cases, No.	Lethal cases, No.	Indolent cases, No.
5	55233305	GCT	G	.	*CCNO*	exonic	deletion	0	0	1	0
5	60928888	T	TC	– [b]	*ERCC8*	exonic	frameshift insertion	0	0	0	1
5	83104942	TC	T	rs757928483	*XRCC4*	exonic	deletion	0	0	1	0
5	83258613	C	T	– [b]	*XRCC4*	exonic	stopgain	0	0	1	0
5	132579486	A	AT	rs786201531	*RAD50*	exonic	frameshift insertion	0	0	0	1
5	132579907	AG	A	– [b]	*RAD50*	exonic	deletion	0	0	1	0
5	132642203	CG	C	rs786202259	*RAD50*	exonic	deletion	0	0	1	0
5	176968670	T	TGC	– [b]	*UIMC1*	exonic	frameshift insertion	1	0	0	0
6	43582314	A	G	– [b]	*POLH*	splicing	– [b]	0	0	1	0
6	43610707	G	T	– [b]	*POLH*	exonic	stopgain	0	0	1	0
7	5995572	ACTGT	A	rs267608154	*PMS2*	exonic	deletion	0	0	1	0
7	6002670	G	C	rs200029834	*PMS2*	exonic	stopgain	0	0	1	0
8	89964509	T	A	– [b]	*NBN*	splicing	– [b]	1	0	0	0
8	94378209	C	T	– [b]	*RAD54B*	exonic	nonsynonymous SNV	0	0	1	0
8	94391872	C	T	rs115960331	*RAD54B*	exonic	nonsynonymous SNV	1	0	0	0
8	94399497	T	G	rs768725152	*RAD54B*	exonic	nonsynonymous SNV	0	0	0	1
8	94400413	T	C	– [b]	*RAD54B*	exonic	nonsynonymous SNV	1	0	0	0
8	144513108	CAT	C	rs752729755	*RECQL4*	exonic	deletion	1	0	1	0
8	144513126	G	A	rs386833851	*RECQL4*	exonic	stopgain	0	0	1	0
8	144514982	CA	C	rs386833845	*RECQL4*	exonic	deletion	0	0	2	1
8	144517482	TCA	T	– [b]	*RECQL4*	exonic	deletion	0	0	0	1
9	32989864	G	A	– [b]	*APTX*	exonic	stopgain	0	0	0	1
9	35059649	A	AT	– [b]	*VCP*	exonic	frameshift insertion	0	1	0	0
9	97675528	C	CA	– [b]	*XPA*	exonic	stopgain	0	0	1	0
10	14908497	G	A	rs200693133	*DCLRE1C*	exonic	stopgain	0	0	0	1

CHROM[a]	POS	REF	ALT	RS_ID	Ref. Gene	Function	Exonic function	African American		European American	
								Lethal cases, No.	Indolent cases, No.	Lethal cases, No.	Indolent cases, No.
10	72102414	T	C	– [b]	*ASCC1*	splicing	– [b]	1	0	0	0
11	47235354	GC	G	– [b]	*DDB2*	exonic	deletion	0	0	1	0
11	94470470	C	T	– [b]	*MRE11*	splicing	– [b]	0	1	0	0
11	108251025	CAG	C	rs751357509	*ATM*	exonic	deletion	0	0	1	0
11	108281067	G	GCTGTGGTTTTATC	– [b]	*ATM*	exonic	frameshift insertion	0	0	1	0
11	108317400	AT	A	rs786203008	*ATM*	exonic	deletion	0	0	1	0
11	108332886	G	A	rs377349459	*ATM*	exonic	stopgain	1	0	0	0
12	109098458	TG	T	rs759483250	*UNG*	exonic	deletion	0	1	0	0
12	132642699	C	A	– [b]	*POLE*	exonic	nonsynonymous SNV	0	0	0	1
13	32319154	G	T	rs80358435	*BRCA2*	exonic	stopgain	0	0	3	0
13	32332614	GA	G	rs80359264	*BRCA2*	exonic	deletion	0	0	1	0
13	32332667	C	CTTAG	rs397515635	*BRCA2*	exonic	frameshift insertion	0	0	1	0
13	32337035	GT	G	– [b]	*BRCA2*	exonic	deletion	0	0	1	0
13	32337189	AAG	A	rs80359357	*BRCA2*	exonic	deletion	0	0	1	0
13	32338200	CTG	C	rs746229647	*BRCA2*	exonic	deletion	0	0	1	0
13	32338988	CT	C	rs80359462	*BRCA2*	exonic	deletion	0	0	1	0
13	32340072	ACT	A	rs80359530	*BRCA2*	exonic	deletion	0	0	1	0
13	32340300	GT	G	rs80359550	*BRCA2*	exonic	deletion	0	0	2	0
13	32356550	C	T	rs80358981	*BRCA2*	exonic	stopgain	1	0	0	0
13	32371000	AAG	A	rs80359714	*BRCA2*	exonic	deletion	0	0	3	0
13	102846279	G	A	rs756544378	*ERCC5*	exonic	nonsynonymous SNV	0	0	1	0
13	102866765	C	T	rs774078839	*ERCC5*	exonic	nonsynonymous SNV	0	0	0	1
14	75017123	G	A	rs148895027	*MLH3*	exonic	stopgain	0	0	0	1
14	75047858	G	A	– [b]	*MLH3*	exonic	stopgain	1	0	0	0
15	40731103	C	T	rs139810756	*RAD51*	exonic	stopgain	0	1	0	0
15	89274167	G	T	– [b]	*FANCI*	splicing	– [b]	0	0	0	1
15	89317389	G	GTATC	– [b]	*POLG*	exonic	stopgain	1	0	0	0

CHROM[a]	POS	REF	ALT	RS_ID	Ref. Gene	Function	Exonic function	African American		European American	
								Lethal cases, No.	Indolent cases, No.	Lethal cases, No.	Indolent cases, No.
15	90750035	ACT	A	rs760209332	*BLM*	exonic	deletion	0	0	1	0
15	90766926	T	TA	rs758886088	*BLM*	exonic	frameshift insertion	0	0	1	1
16	2040004	G	A	rs146347092	*NTHL1*	exonic	stopgain	0	1	1	0
16	2043727	C	T	rs779757251	*NTHL1*	splicing	– [b]	0	0	0	1
16	2047708	C	T	rs749908882	*NTHL1*	splicing	– [b]	0	0	1	1
16	3589378	T	TG	rs781663547	*SLX4*	exonic	frameshift insertion	0	0	1	0
16	3594476	G	A	rs760126773	*SLX4*	exonic	stopgain	0	0	1	0
16	89816551	C	T	rs761341952	*FANCA*	exonic	stopgain	0	0	1	0
17	7670711	CG	C	– [b]	*TP53*	exonic	deletion	0	0	0	1
17	7675208	CA	C	– [b]	*TP53*	exonic	deletion	0	0	1	0
17	58734199	C	T	rs756744016	*RAD51C*	exonic	stopgain	1	0	0	0
17	61799232	C	G	– [b]	*BRIP1*	exonic	nonsynonymous SNV	0	0	0	1
17	61801265	TTG	T	rs587780224	*BRIP1*	exonic	deletion	1	0	0	0
17	61808495	T	C	rs28997570	*BRIP1*	exonic	nonsynonymous SNV	0	1	0	0
18	22992797	C	T	rs373907735	*RBBP8*	exonic	stopgain	0	0	1	0
19	43543449	G	GC	rs773401514	*XRCC1*	exonic	frameshift insertion	0	0	0	1
19	45352780	A	AC	rs756630156	*ERCC2*	exonic	frameshift insertion	0	0	1	0
19	45353295	TAA	T	rs587778271	*ERCC2*	exonic	deletion	0	0	1	0
19	45354841	CCGGATCACAGCTGCAA	C	– [b]	*ERCC2*	exonic	deletion	1	0	0	0
19	45365053	G	A	rs151235136	*ERCC2*	exonic	stopgain	0	0	0	1
19	49861800	T	TGTTGTCGATGGCGACCC	rs587784365	*PNKP*	exonic	frameshift insertion	0	0	0	1

[a] Chromosome and position based on human genome build 38 (GRCh38).

[b] Not available/applicable.

Supplementary Table 2. Carrier frequencies for pathogenic sequence variants by DNA damage response gene.

DDR gene	All men				African American				European American			
	Lethal cases, No. (%)			Indolent cases, No. (%)	Lethal cases, No. (%)			Indolent cases, No. (%)	Lethal cases, No. (%)			Indolent cases, No. (%)
	All	Diagnosed < age 65[a]	Diagnosed ≥ age 65[a]		All	Diagnosed < age 65[a]	Diagnosed ≥ age 65[a]		All	Diagnosed < age 65[a]	Diagnosed ≥ age 65[a]	
APTX	0 (0.0)	0 (0.0)	0 (0.0)	1 (0.3)	0 (0.0)	0 (0.0)	0 (0.0)	0 (0.0)	0 (0.0)	0 (0.0)	0 (0.0)	1 (0.4)
ASCC1	1 (0.2)	1 (0.4)	0 (0.0)	0 (0.0)	1 (0.9)	1 (1.6)	0 (0.0)	0 (0.0)	0 (0.0)	0 (0.0)	0 (0.0)	0 (0.0)
ATM	4 (1.0)	3 (1.2)	1 (0.6)	0 (0.0)	1 (0.9)	1 (1.6)	0 (0.0)	0 (0.0)	3 (1.0)	2 (1.1)	1 (0.9)	0 (0.0)
ATR	1 (0.2)	1 (0.4)	0 (0.0)	0 (0.0)	1 (0.9)	1 (1.6)	0 (0.0)	0 (0.0)	0 (0.0)	0 (0.0)	0 (0.0)	0 (0.0)
BARD1	2 (0.5)	1 (0.4)	1 (0.6)	0 (0.0)	0 (0.0)	0 (0.0)	0 (0.0)	0 (0.0)	2 (0.7)	1 (0.6)	1 (0.9)	0 (0.0)
BLM	2 (0.5)	2 (0.8)	0 (0.0)	1 (0.3)	0 (0.0)	0 (0.0)	0 (0.0)	0 (0.0)	2 (0.7)	2 (1.1)	0 (0.0)	1 (0.4)
BRCA2	16 (3.9)	11 (4.5)	5 (3.2)	0 (0.0)	1 (0.9)	1 (1.6)	0 (0.0)	0 (0.0)	15 (5.1)	10 (5.6)	5 (4.4)	0 (0.0)
BRIP1	1 (0.2)	1 (0.4)	0 (0.0)	2 (0.6)	1 (0.9)	1 (1.6)	0 (0.0)	1 (1.4)	0 (0.0)	0 (0.0)	0 (0.0)	1 (0.4)
CCNO	3 (0.7)	3 (1.2)	0 (0.0)	0 (0.0)	0 (0.0)	0 (0.0)	0 (0.0)	0 (0.0)	3 (1.0)	3 (1.7)	0 (0.0)	0 (0.0)
DCLRE1C	0 (0.0)	0 (0.0)	0 (0.0)	1 (0.3)	0 (0.0)	0 (0.0)	0 (0.0)	0 (0.0)	0 (0.0)	0 (0.0)	0 (0.0)	1 (0.4)
DDB2	1 (0.2)	1 (0.4)	0 (0.0)	0 (0.0)	0 (0.0)	0 (0.0)	0 (0.0)	0 (0.0)	1 (0.3)	1 (0.6)	0 (0.0)	0 (0.0)
ERCC2	3 (0.7)	1 (0.4)	2 (1.3)	1 (0.3)	1 (0.9)	0 (0.0)	1 (2.4)	0 (0.0)	2 (0.7)	1 (0.6)	1 (0.9)	1 (0.4)
ERCC3	2 (0.5)	2 (0.8)	0 (0.0)	2 (0.6)	1 (0.9)	1 (1.6)	0 (0.0)	0 (0.0)	1 (0.3)	1 (0.6)	0 (0.0)	2 (0.7)
ERCC5	1 (0.2)	0 (0.0)	1 (0.6)	1 (0.3)	0 (0.0)	0 (0.0)	0 (0.0)	0 (0.0)	1 (0.3)	0 (0.0)	1 (0.9)	1 (0.4)
ERCC8	0 (0.0)	0 (0.0)	0 (0.0)	1 (0.3)	0 (0.0)	0 (0.0)	0 (0.0)	0 (0.0)	0 (0.0)	0 (0.0)	0 (0.0)	1 (0.4)
EXO1	0 (0.0)	0 (0.0)	0 (0.0)	1 (0.3)	0 (0.0)	0 (0.0)	0 (0.0)	0 (0.0)	0 (0.0)	0 (0.0)	0 (0.0)	1 (0.4)
FANCA	1 (0.2)	0 (0.0)	1 (0.6)	0 (0.0)	0 (0.0)	0 (0.0)	0 (0.0)	0 (0.0)	1 (0.3)	0 (0.0)	1 (0.9)	0 (0.0)
FANCI	0 (0.0)	0 (0.0)	0 (0.0)	1 (0.3)	0 (0.0)	0 (0.0)	0 (0.0)	0 (0.0)	0 (0.0)	0 (0.0)	0 (0.0)	1 (0.4)
MLH1	0 (0.0)	0 (0.0)	0 (0.0)	1 (0.3)	0 (0.0)	0 (0.0)	0 (0.0)	0 (0.0)	0 (0.0)	0 (0.0)	0 (0.0)	1 (0.4)
MLH3	1 (0.2)	0 (0.0)	1 (0.6)	1 (0.3)	1 (0.9)	0 (0.0)	1 (2.4)	0 (0.0)	0 (0.0)	0 (0.0)	0 (0.0)	1 (0.4)
MRE11	0 (0.0)	0 (0.0)	0 (0.0)	1 (0.3)	0 (0.0)	0 (0.0)	0 (0.0)	1 (1.4)	0 (0.0)	0 (0.0)	0 (0.0)	0 (0.0)
MSH2	4 (1.0)	3 (1.2)	1 (0.6)	1 (0.3)	2 (1.8)	1 (1.6)	1 (2.4)	0 (0.0)	2 (0.7)	2 (1.1)	0 (0.0)	1 (0.4)
MSH6	1 (0.2)	0 (0.0)	1 (0.6)	0 (0.0)	0 (0.0)	0 (0.0)	0 (0.0)	0 (0.0)	1 (0.3)	0 (0.0)	1 (0.9)	0 (0.0)
MUTYH	1 (0.2)	0 (0.0)	1 (0.6)	0 (0.0)	1 (0.9)	0 (0.0)	1 (2.4)	0 (0.0)	0 (0.0)	0 (0.0)	0 (0.0)	0 (0.0)
NBN	1 (0.2)	0 (0.0)	1 (0.6)	0 (0.0)	1 (0.9)	0 (0.0)	1 (2.4)	0 (0.0)	0 (0.0)	0 (0.0)	0 (0.0)	0 (0.0)

NTHL1	2 (0.5)	2 (0.8)	0 (0.0)	3 (0.8)	0 (0.0)	0 (0.0)	0 (0.0)	1 (1.4)	2 (0.7)	2 (1.1)	0 (0.0)	2 (0.7)
PER2	1 (0.2)	0 (0.0)	1 (0.6)	0 (0.0)	1 (0.9)	0 (0.0)	1 (2.4)	0 (0.0)	0 (0.0)	0 (0.0)	0 (0.0)	0 (0.0)
PMS2	2 (0.5)	0 (0.0)	2 (1.3)	0 (0.0)	0 (0.0)	0 (0.0)	0 (0.0)	0 (0.0)	2 (0.7)	0 (0.0)	2 (1.8)	0 (0.0)
PNKP	0 (0.0)	0 (0.0)	0 (0.0)	1 (0.3)	0 (0.0)	0 (0.0)	0 (0.0)	0 (0.0)	0 (0.0)	0 (0.0)	0 (0.0)	1 (0.4)
POLE	0 (0.0)	0 (0.0)	0 (0.0)	1 (0.3)	0 (0.0)	0 (0.0)	0 (0.0)	0 (0.0)	0 (0.0)	0 (0.0)	0 (0.0)	1 (0.4)
POLG	1 (0.2)	0 (0.0)	1 (0.6)	0 (0.0)	1 (0.9)	0 (0.0)	1 (2.4)	0 (0.0)	0 (0.0)	0 (0.0)	0 (0.0)	0 (0.0)
POLH	2 (0.5)	2 (0.8)	0 (0.0)	0 (0.0)	0 (0.0)	0 (0.0)	0 (0.0)	0 (0.0)	2 (0.7)	2 (1.1)	0 (0.0)	0 (0.0)
RAD50	2 (0.5)	2 (0.8)	0 (0.0)	1 (0.3)	0 (0.0)	0 (0.0)	0 (0.0)	0 (0.0)	2 (0.7)	2 (1.1)	0 (0.0)	1 (0.4)
RAD51	0 (0.0)	0 (0.0)	0 (0.0)	1 (0.3)	0 (0.0)	0 (0.0)	0 (0.0)	1 (1.4)	0 (0.0)	0 (0.0)	0 (0.0)	0 (0.0)
RAD51C	1 (0.2)	1 (0.4)	0 (0.0)	0 (0.0)	1 (0.9)	1 (1.6)	0 (0.0)	0 (0.0)	0 (0.0)	0 (0.0)	0 (0.0)	0 (0.0)
RAD54B	3 (0.7)	1 (0.4)	2 (1.3)	1 (0.3)	2 (1.8)	0 (0.0)	2 (4.8)	0 (0.0)	1 (0.3)	1 (0.6)	0 (0.0)	1 (0.4)
RAD54L	4 (1.0)	4 (1.7)	0 (0.0)	6 (1.7)	0 (0.0)	0 (0.0)	0 (0.0)	2 (2.7)	4 (1.4)	4 (2.2)	0 (0.0)	4 (1.4)
RBBP8	1 (0.2)	0 (0.0)	1 (0.6)	0 (0.0)	0 (0.0)	0 (0.0)	0 (0.0)	0 (0.0)	1 (0.3)	0 (0.0)	1 (0.9)	0 (0.0)
RECQL4	5 (1.2)	3 (1.2)	2 (1.3)	2 (0.6)	1 (0.9)	1 (1.6)	0 (0.0)	0 (0.0)	4 (1.4)	2 (1.1)	2 (1.8)	2 (0.7)
SLX4	2 (0.5)	1 (0.4)	1 (0.6)	0 (0.0)	0 (0.0)	0 (0.0)	0 (0.0)	0 (0.0)	2 (0.7)	1 (0.6)	1 (0.9)	0 (0.0)
TP53	1 (0.2)	1 (0.4)	0 (0.0)	1 (0.3)	0 (0.0)	0 (0.0)	0 (0.0)	0 (0.0)	1 (0.3)	1 (0.6)	0 (0.0)	1 (0.4)
UIMC1	1 (0.2)	0 (0.0)	0 (0.0)	0 (0.0)	1 (0.9)	0 (0.0)	0 (0.0)	0 (0.0)	0 (0.0)	0 (0.0)	0 (0.0)	0 (0.0)
UNG	0 (0.0)	0 (0.0)	0 (0.0)	1 (0.3)	0 (0.0)	0 (0.0)	0 (0.0)	1 (1.4)	0 (0.0)	0 (0.0)	0 (0.0)	0 (0.0)
VCP	0 (0.0)	0 (0.0)	0 (0.0)	1 (0.3)	0 (0.0)	0 (0.0)	0 (0.0)	1 (1.4)	0 (0.0)	0 (0.0)	0 (0.0)	0 (0.0)
XPA	1 (0.2)	1 (0.4)	0 (0.0)	0 (0.0)	0 (0.0)	0 (0.0)	0 (0.0)	0 (0.0)	1 (0.3)	1 (0.6)	0 (0.0)	0 (0.0)
XRCC1	0 (0.0)	0 (0.0)	0 (0.0)	1 (0.3)	0 (0.0)	0 (0.0)	0 (0.0)	0 (0.0)	0 (0.0)	0 (0.0)	0 (0.0)	1 (0.4)
XRCC4	2 (0.5)	1 (0.4)	1 (0.6)	0 (0.0)	0 (0.0)	0 (0.0)	0 (0.0)	0 (0.0)	2 (0.7)	1 (0.6)	1 (0.9)	0 (0.0)

[a] Numbers may not add up because date of diagnosis was not available for all lethal cases. DDR = DNA damage response.

Supplementary Table 3. Association results by DNA damage response pathway and age at diagnosis.

Pathway	All men				African American				European American			
	Lethal cases, No. (%)		Indolent cases, No. (%)	P^b	Lethal cases, No. (%)		Indolent cases, No. (%)	P^b	Lethal cases, No. (%)		Indolent cases, No. (%)	P^b
	Diagnosed < age 65[a]	Diagnosed ≥ age 65[a]			Diagnosed < age 65[a]	Diagnosed ≥ age 65[a]			Diagnosed < age 65[a]	Diagnosed ≥ age 65[a]		
Any DDR pathway	48 (19.8)	27 (17.3)	34 (9.6)	6.30×10^{-4}	9 (14.5)	9 (21.4)	7 (9.5)	0.43	39 (21.7)	18 (15.8)	27 (9.6)	5.70×10^{-4}
Core pathways	44 (18.2)	24 (15.4)	32 (9.0)	0.002	8 (12.9)	7 (16.7)	6 (8.1)	0.41	36 (20.0)	17 (14.9)	26 (9.3)	0.001
HR	25 (10.3)	11 (7.1)	13 (3.7)	0.002	3 (4.8)	3 (7.1)	4 (5.4)	1.00	22 (12.2)	8 (7.0)	9 (3.2)	2.40×10^{-4}
FA	16 (6.6)	7 (4.5)	5 (1.4)	0.001	3 (4.8)	0 (0.0)	2 (2.7)	0.66	13 (7.2)	7 (6.1)	3 (1.1)	9.00×10^{-4}
NHEJ	6 (2.5)	3 (1.9)	6 (1.7)	0.56	1 (1.6)	1 (2.4)	1 (1.4)	1.00	5 (2.8)	2 (1.8)	5 (1.8)	0.52
MMR	3 (1.2)	5 (3.2)	4 (1.1)	1.00	1 (1.6)	2 (4.8)	0 (0.0)	0.46	2 (1.1)	3 (2.6)	4 (1.4)	1.00
NER	5 (2.1)	3 (1.9)	6 (1.7)	0.76	1 (1.6)	1 (2.4)	0 (0.0)	0.46	4 (2.2)	2 (1.8)	6 (2.1)	1.00
BER	4 (1.7)	1 (0.6)	8 (2.3)	0.77	0 (0.0)	1 (2.4)	2 (2.7)	0.50	4 (2.2)	0 (0.0)	6 (2.1)	1.00
TLS	2 (0.8)	0 (0.0)	0 (0.0)	0.16	0 (0.0)	0 (0.0)	0 (0.0)	– [c]	2 (1.1)	0 (0.0)	0 (0.0)	0.15
DR	1 (0.4)	0 (0.0)	0 (0.0)	0.41	1 (1.6)	0 (0.0)	0 (0.0)	0.46	0 (0.0)	0 (0.0)	0 (0.0)	– [c]
Non-core pathways	12 (5.0)	5 (3.2)	5 (1.4)	0.02	2 (3.2)	3 (7.1)	2 (2.7)	1.00	10 (5.6)	2 (1.8)	3 (1.1)	0.007
Check-point factor	6 (2.5)	3 (1.9)	1 (0.3)	0.02	2 (3.2)	1 (2.4)	0 (0.0)	0.21	4 (2.2)	2 (1.8)	1 (0.4)	0.08
Other non-core	5 (2.1)	1 (0.6)	4 (1.1)	0.50	0 (0.0)	1 (2.4)	1 (1.4)	1.00	5 (2.8)	0 (0.0)	3 (1.1)	0.27
Probable associated	3 (1.2)	1 (0.6)	1 (0.3)	0.31	0 (0.0)	1 (2.4)	1 (1.4)	1.00	3 (1.7)	0 (0.0)	0 (0.0)	0.06

[a] Numbers may not add up because date of diagnosis was not available for all lethal cases. DDR = DNA damage response; HR = homologous recombination; FA= Fanconi anemia; NHEJ = non-homologous end joining; MMR = mismatch repair; NER = nucleotide excision repair; BER = base excision repair; TLS = translesion synthesis; DR = direct repair.

[b] P-values from two-sided Fisher's exact test comparing lethal cases diagnosed < age 65 years against indolent cases.

[c] Not applicable.

Supplementary Table 4. Association results by DNA damage response pathway and age at lethal event.

Pathway	All men				African American				European American			
	Lethal cases, No. (%)		Indolent cases, No. (%)	P^b	Lethal cases, No. (%)		Indolent cases, No. (%)	P^b	Lethal cases, No. (%)		Indolent cases, No. (%)	P^b
	Lethal event < age 65[a]	Lethal event ≥ age 65[a]			Lethal event < age 65[a]	Lethal event ≥ age 65[a]			Lethal event < age 65[a]	Lethal event ≥ age 65[a]		
Any DDR pathway	34 (20.9)	40 (17.9)	34 (9.6)	7.00×10^{-4}	6 (17.6)	11 (19.0)	7 (9.5)	0.34	28 (21.7)	29 (17.5)	27 (9.6)	0.002
Core pathways	31 (19.0)	36 (16.1)	32 (9.0)	0.002	5 (14.7)	9 (15.5)	6 (8.1)	0.32	26 (20.2)	27 (16.3)	26 (9.3)	0.004
HR	17 (10.4)	18 (8.0)	13 (3.7)	0.004	2 (5.9)	3 (5.2)	4 (5.4)	1.00	15 (11.6)	15 (9.0)	9 (3.2)	0.002
FA	12 (7.4)	10 (4.5)	5 (1.4)	8.90×10^{-4}	2 (5.9)	0 (0.0)	2 (2.7)	0.59	10 (7.8)	10 (6.0)	3 (1.1)	8.40×10^{-4}
NHEJ	5 (3.1)	4 (1.8)	6 (1.7)	0.33	1 (2.9)	1 (1.7)	1 (1.4)	0.53	4 (3.1)	3 (1.8)	5 (1.8)	0.47
MMR	2 (1.2)	6 (2.7)	4 (1.1)	1.00	1 (2.9)	2 (3.4)	0 (0.0)	0.31	1 (0.8)	4 (2.4)	4 (1.4)	1.00
NER	3 (1.8)	5 (2.2)	6 (1.7)	1.00	0 (0.0)	2 (3.4)	0 (0.0)	– [c]	3 (2.3)	3 (1.8)	6 (2.1)	1.00
BER	3 (1.8)	2 (0.9)	8 (2.3)	1.00	0 (0.0)	1 (1.7)	2 (2.7)	1.00	3 (2.3)	1 (0.6)	6 (2.1)	1.00
TLS	1 (0.6)	1 (0.4)	0 (0.0)	0.32	0 (0.0)	0 (0.0)	0 (0.0)	– [c]	1 (0.8)	1 (0.6)	0 (0.0)	0.32
DR	0 (0.0)	1 (0.4)	0 (0.0)	– [c]	0 (0.0)	1 (1.7)	0 (0.0)	– [c]	0 (0.0)	0 (0.0)	0 (0.0)	– [c]
Non-core pathways	9 (5.5)	8 (3.6)	5 (1.4)	0.02	2 (5.9)	3 (5.2)	2 (2.7)	0.59	7 (5.4)	5 (3.0)	3 (1.1)	0.01
Check-point factor	5 (3.1)	4 (1.8)	1 (0.3)	0.01	2 (5.9)	1 (1.7)	0 (0.0)	0.10	3 (2.3)	3 (1.8)	1 (0.4)	0.10
Other non-core	3 (1.8)	3 (1.3)	4 (1.1)	0.68	0 (0.0)	1 (1.7)	1 (1.4)	1.00	3 (2.3)	2 (1.2)	3 (1.1)	0.38
Probable associated	2 (1.2)	2 (0.9)	1 (0.3)	0.24	0 (0.0)	1 (1.7)	1 (1.4)	1.00	2 (1.6)	1 (0.6)	0 (0.0)	0.10

[a] Numbers may not add up because date of lethal disease was not available for all lethal cases. DDR = DNA damage response; HR = homologous recombination; FA= Fanconi anemia; NHEJ = non-homologous end joining; MMR = mismatch repair; NER = nucleotide excision repair; BER = base excision repair; TLS = translesion synthesis; DR = direct repair.

[b] P-values from two-sided Fisher's exact test comparing lethal cases with a lethal event < age 65 years against indolent cases.

[c] Not applicable.

Supplementary Table 5. Association results by DNA damage response pathway excluding *BRCA2*.

Pathway	All men			African American			European American		
	Lethal cases, No. (%)	Indolent cases, No. (%)	P^a	Lethal cases, No. (%)	Indolent cases, No. (%)	P^a	Lethal cases, No. (%)	Indolent cases, No. (%)	P^a
Any DDR pathway	60 (14.6)	34 (9.6)	0.04	18 (15.8)	7 (9.5)	0.27	42 (14.2)	27 (9.6)	0.10
Core pathways	53 (12.9)	32 (9.0)	0.11	15 (13.2)	6 (8.1)	0.35	38 (12.8)	26 (9.3)	0.19
HR	21 (5.1)	13 (3.7)	0.38	6 (5.3)	4 (5.4)	1.00	15 (5.1)	9 (3.2)	0.30
FA	7 (1.7)	5 (1.4)	0.78	2 (1.8)	2 (2.7)	0.65	5 (1.7)	3 (1.1)	0.73
NHEJ	9 (2.2)	6 (1.7)	0.80	2 (1.8)	1 (1.4)	1.00	7 (2.4)	5 (1.8)	0.77
MMR	8 (2.0)	4 (1.1)	0.40	3 (2.6)	0 (0.0)	0.28	5 (1.7)	4 (1.4)	1.00
NER	8 (2.0)	6 (1.7)	1.00	2 (1.8)	0 (0.0)	0.52	6 (2.0)	6 (2.1)	1.00
BER	5 (1.2)	8 (2.3)	0.40	1 (0.9)	2 (2.7)	0.56	4 (1.4)	6 (2.1)	0.54
TLS	2 (0.5)	0 (0.0)	0.50	0 (0.0)	0 (0.0)	– [b]	2 (0.7)	0 (0.0)	0.50
DR	1 (0.2)	0 (0.0)	1.00	1 (0.9)	0 (0.0)	1.00	0 (0.0)	0 (0.0)	– [b]
Non-core pathways	17 (4.1)	5 (1.4)	0.03	5 (4.4)	2 (2.7)	0.71	12 (4.1)	3 (1.1)	0.03
Checkpoint factor	9 (2.2)	1 (0.3)	0.02	3 (2.6)	0 (0.0)	0.28	6 (2.0)	1 (0.4)	0.12
Other non-core	6 (1.5)	4 (1.1)	0.76	1 (0.9)	1 (1.4)	1.00	5 (1.7)	3 (1.1)	0.73
Probable associated	4 (1.0)	1 (0.3)	0.38	1 (0.9)	1 (1.4)	1.00	3 (1.0)	0 (0.0)	0.25

[a] P-values from two-sided Fisher's exact test comparing lethal cases against indolent cases. DDR = DNA damage response; HR = homologous recombination; FA= Fanconi anemia; NHEJ = non-homologous end joining; MMR = mismatch repair; NER = nucleotide excision repair; BER = base excision repair; TLS = translesion synthesis; DR = direct repair.
[b] Not applicable.

Supplementary Table 6. Association results by DNA damage response pathway including only genes by Pritchard et al.

Pathway	All men			African American			European American		
	Lethal cases, No. (%)	Indolent cases, No. (%)	P^a	Lethal cases, No. (%)	Indolent cases, No. (%)	P^a	Lethal cases, No. (%)	Indolent cases, No. (%)	P^a
Any DDR pathway[b]	33 (8.0)	4 (1.1)	4.40×10^{-6}	8 (7.0)	1 (1.4)	0.09	25 (8.4)	3 (1.1)	2.20×10^{-5}
Core pathways	32 (7.8)	4 (1.1)	7.80×10^{-6}	7 (6.1)	1 (1.4)	0.15	25 (8.4)	3 (1.1)	2.20×10^{-5}
HR	18 (4.4)	0 (0.0)	1.20×10^{-5}	3 (2.6)	0 (0.0)	0.28	15 (5.1)	0 (0.0)	5.50×10^{-5}
FA	20 (4.9)	2 (0.6)	2.80×10^{-4}	3 (2.6)	1 (1.4)	1.00	17 (5.7)	1 (0.4)	1.30×10^{-4}
NHEJ	5 (1.2)	0 (0.0)	0.07	2 (1.8)	0 (0.0)	0.52	3 (1.0)	0 (0.0)	0.25
MMR	7 (1.7)	2 (0.6)	0.19	2 (1.8)	0 (0.0)	0.52	5 (1.7)	2 (0.7)	0.45
NER	0 (0.0)	0 (0.0)	– [c]	0 (0.0)	0 (0.0)	– [c]	0 (0.0)	0 (0.0)	– [c]
BER	0 (0.0)	0 (0.0)	– [c]	0 (0.0)	0 (0.0)	– [c]	0 (0.0)	0 (0.0)	– [c]
TLS	0 (0.0)	0 (0.0)	– [c]	0 (0.0)	0 (0.0)	– [c]	0 (0.0)	0 (0.0)	– [c]
DR	0 (0.0)	0 (0.0)	– [c]	0 (0.0)	0 (0.0)	– [c]	0 (0.0)	0 (0.0)	– [c]
Non-core pathways	6 (1.5)	0 (0.0)	0.03	3 (2.6)	0 (0.0)	0.28	3 (1.0)	0 (0.0)	0.25
Checkpoint factor	5 (1.2)	0 (0.0)	0.07	2 (1.8)	0 (0.0)	0.52	3 (1.0)	0 (0.0)	0.25
Other non-core	1 (0.2)	0 (0.0)	1.00	1 (0.9)	0 (0.0)	1.00	0 (0.0)	0 (0.0)	– [c]
Probable associated	0 (0.0)	0 (0.0)	– [c]	0 (0.0)	0 (0.0)	– [c]	0 (0.0)	0 (0.0)	– [c]

[a] P-values from two-sided Fisher's exact test comparing lethal cases against indolent cases. DDR = DNA damage response; HR = homologous recombination; FA= Fanconi anemia; NHEJ = non-homologous end joining; MMR = mismatch repair; NER = nucleotide excision repair; BER = base excision repair; TLS = translesion synthesis; DR = direct repair.

[b] The following genes from Pritchard et al. were included: *ATM*, *ATR*, *BARD1*, *BRCA1*, *BRCA2*, *BRIP1*, *CHEK2*, *MLH1*, *MRE11A*, *MSH2*, *MSH6*, *NBN*, *PALB2*, *PMS2*, R*AD51C*, *RAD51D*, *XRCC2* (not sequenced: *BAP1*, *FAM175A*, *GEN1*).

[c] Not applicable.

Supplementary Table 7. Association results by DNA damage response pathway excluding *RAD54L*.

	All men			African American			European American		
Pathway	Lethal cases, No. (%)	Indolent cases, No. (%)	P^a	Lethal cases, No. (%)	Indolent cases, No. (%)	P^a	Lethal cases, No. (%)	Indolent cases, No. (%)	P^a
Any DDR pathway	72 (17.6)	28 (7.9)	9.30×10^{-5}	19 (16.7)	5 (6.8)	0.07	53 (17.9)	23 (8.2)	8.00×10^{-4}
Core pathways	65 (15.9)	26 (7.3)	3.10×10^{-4}	16 (14.0)	4 (5.4)	0.09	49 (16.6)	22 (7.9)	0.002
HR	33 (8.0)	7 (2.0)	1.30×10^{-4}	7 (6.1)	2 (2.7)	0.49	26 (8.8)	5 (1.8)	1.50×10^{-4}
FA	23 (5.6)	5 (1.4)	0.002	3 (2.6)	2 (2.7)	1.00	20 (6.8)	3 (1.1)	4.20×10^{-4}
NHEJ	9 (2.2)	6 (1.7)	0.80	2 (1.8)	1 (1.4)	1.00	7 (2.4)	5 (1.8)	0.77
MMR	8 (2.0)	4 (1.1)	0.40	3 (2.6)	0 (0.0)	0.28	5 (1.7)	4 (1.4)	1.00
NER	8 (2.0)	6 (1.7)	1.00	2 (1.8)	0 (0.0)	0.52	6 (2.0)	6 (2.1)	1.00
BER	5 (1.2)	8 (2.3)	0.40	1 (0.9)	2 (2.7)	0.56	4 (1.4)	6 (2.1)	0.54
TLS	2 (0.5)	0 (0.0)	0.50	0 (0.0)	0 (0.0)	– [b]	2 (0.7)	0 (0.0)	0.50
DR	1 (0.2)	0 (0.0)	1.00	1 (0.9)	0 (0.0)	1.00	0 (0.0)	0 (0.0)	– [b]
Non-core pathways	17 (4.1)	5 (1.4)	0.03	5 (4.4)	2 (2.7)	0.71	12 (4.1)	3 (1.1)	0.03
Checkpoint factor	9 (2.2)	1 (0.3)	0.02	3 (2.6)	0 (0.0)	0.28	6 (2.0)	1 (0.4)	0.12
Other non-core	6 (1.5)	4 (1.1)	0.76	1 (0.9)	1 (1.4)	1.00	5 (1.7)	3 (1.1)	0.73
Probable associated	4 (1.0)	1 (0.3)	0.38	1 (0.9)	1 (1.4)	1.00	3 (1.0)	0 (0.0)	0.25

[a] P-values from two-sided Fisher's exact test comparing lethal cases against indolent cases. DDR = DNA damage response; HR = homologous recombination; FA= Fanconi anemia; NHEJ = non-homologous end joining; MMR = mismatch repair; NER = nucleotide excision repair; BER = base excision repair; TLS = translesion synthesis; DR = direct repair.

[b] Not applicable.

Supplementary Table 8. Association results for the most likely pathogenic variants among the variants of unknown clinical significance (VUS) by DNA damage response pathway.

	All men			African American			European American		
Pathway	Lethal cases, No. (%)	Indolent cases, No. (%)	*P*[a]	Lethal cases, No. (%)	Indolent cases, No. (%)	*P*[a]	Lethal cases, No. (%)	Indolent cases, No. (%)	*P*[a]
Any DDR pathway	294 (71.7)	242 (68.4)	0.34	98 (86.0)	56 (75.7)	0.08	196 (66.2)	186 (66.4)	1.00
Core pathways	250 (61.0)	207 (58.5)	0.51	85 (74.6)	51 (68.9)	0.41	165 (55.7)	156 (55.7)	1.00
HR	56 (13.7)	36 (10.2)	0.15	27 (23.7)	17 (23.0)	1.00	29 (9.8)	19 (6.8)	0.23
FA	59 (14.4)	36 (10.2)	0.08	25 (21.9)	17 (23.0)	0.86	34 (11.5)	19 (6.8)	0.06
NHEJ	72 (17.6)	60 (16.9)	0.85	41 (36.0)	20 (27.0)	0.26	31 (10.5)	40 (14.3)	0.20
MMR	65 (15.9)	60 (16.9)	0.70	29 (25.4)	19 (25.7)	1.00	36 (12.2)	41 (14.6)	0.39
NER	80 (19.5)	60 (16.9)	0.40	20 (17.5)	18 (24.3)	0.27	60 (20.3)	42 (15.0)	0.10
BER	74 (18.0)	65 (18.4)	0.93	38 (33.3)	22 (29.7)	0.63	36 (12.2)	43 (15.4)	0.28
TLS	43 (10.5)	38 (10.7)	1.00	8 (7.0)	2 (2.7)	0.32	35 (11.8)	36 (12.9)	0.80
DR	1 (0.2)	0 (0.0)	1.00	0 (0.0)	0 (0.0)	– [b]	1 (0.3)	0 (0.0)	1.00
Non-core pathways	152 (37.1)	119 (33.6)	0.33	59 (51.8)	33 (44.6)	0.37	93 (31.4)	86 (30.7)	0.86
Checkpoint factor	89 (21.7)	54 (15.3)	0.03	35 (30.7)	17 (23.0)	0.32	54 (18.2)	37 (13.2)	0.11
Other non-core	53 (12.9)	44 (12.4)	0.91	22 (19.3)	12 (16.2)	0.70	31 (10.5)	32 (11.4)	0.79
Probable associated	39 (9.5)	32 (9.0)	0.90	16 (14.0)	7 (9.5)	0.49	23 (7.8)	25 (8.9)	0.65

[a] P-values from two-sided Fisher's exact test comparing lethal cases against indolent cases. DDR = DNA damage response; HR = homologous recombination; FA= Fanconi anemia; NHEJ = non-homologous end joining; MMR = mismatch repair; NER = nucleotide excision repair; BER = base excision repair; TLS = translesion synthesis; DR = direct repair.
[b] Not applicable.

Supplementary Table 9. Most likely pathogenic variants among the variants of unknown clinical significance (VUS).

CHROM [a]	POS	REF	ALT	RS_ID	Ref. Gene	Function	Exonic function	African American		European American	
								Lethal cases, No.	Indolent cases, No.	Lethal cases, No.	Indolent cases, No.
1	3732825	G	A	– [b]	*TP73*	exonic	nonsynonymous SNV	0	0	1	0
1	3732904	G	A	rs376429700	*TP73*	exonic	nonsynonymous SNV	1	0	1	0
1	3732924	G	A	rs138694448	*TP73*	exonic	nonsynonymous SNV	0	0	1	0
1	7803737	G	A	rs754761909	*PER3*	exonic	nonsynonymous SNV	0	0	1	1
1	7803754	G	A	rs757458058	*PER3*	exonic	nonsynonymous SNV	0	0	1	0
1	43697936	C	T	– [b]	*KDM4A*	exonic	nonsynonymous SNV	0	0	1	0
1	43704020	G	A	rs143474834	*KDM4A*	exonic	nonsynonymous SNV	0	0	1	2
1	45330526	C	T	rs3219494	*MUTYH*	exonic	nonsynonymous SNV	2	0	0	0
1	45331485	G	T	rs144079536	*MUTYH*	exonic	nonsynonymous SNV	0	0	1	0
1	45332446	G	A	rs200495564	*MUTYH*	exonic	nonsynonymous SNV	0	0	1	0
1	45332479	C	T	rs200165598	*MUTYH*	exonic	nonsynonymous SNV	0	0	0	1
1	45332803	T	C	rs34612342	*MUTYH*	exonic	nonsynonymous SNV	0	0	2	2
1	46250063	C	T	rs530382665	*RAD54L*	exonic	nonsynonymous SNV	0	0	0	1
1	46274607	C	T	rs150315374	*RAD54L*	exonic	nonsynonymous SNV	0	0	1	0
1	113908100	C	G	rs369389853	*DCLRE1B*	exonic	nonsynonymous SNV	0	0	1	0
1	202335649	C	T	rs200130395	*UBE2T*	exonic	nonsynonymous SNV	0	0	1	1
1	202730977	A	C	rs200000027	*KDM5B*	exonic	nonsynonymous SNV	0	0	1	0
1	202746142	C	T	rs144390145	*KDM5B*	exonic	nonsynonymous SNV	0	0	3	7
1	241850571	G	A	– [b]	*EXO1*	exonic	nonsynonymous SNV	0	0	1	0
2	47475082	T	A	rs376044376	*MSH2*	exonic	nonsynonymous SNV	0	1	0	0
2	47480737	G	A	rs63750757	*MSH2*	exonic	nonsynonymous SNV	0	0	0	2
2	47783259	G	T	– [b]	*MSH6*	exonic	nonsynonymous SNV	0	0	1	0
2	47791097	G	T	rs3211299	*MSH6*	exonic	nonsynonymous SNV	1	0	0	2
2	47798867	A	G	rs267608051	*MSH6*	exonic	nonsynonymous SNV	0	0	0	1
2	47798882	G	A	rs55760494	*MSH6*	exonic	nonsynonymous SNV	0	0	1	0

CHROM [a]	POS	REF	ALT	RS_ID	Ref. Gene	Function	Exonic function	African American		European American	
								Lethal cases, No.	Indolent cases, No.	Lethal cases, No.	Indolent cases, No.
2	47799002	T	C	rs61753793	*MSH6*	exonic	nonsynonymous SNV	0	0	0	1
2	47799386	G	C	– [b]	*MSH6*	exonic	nonsynonymous SNV	0	0	1	0
2	47799509	T	C	rs63751005	*MSH6*	exonic	nonsynonymous SNV	0	0	1	1
2	47800439	A	G	– [b]	*MSH6*	exonic	nonsynonymous SNV	1	0	0	0
2	47800514	T	C	– [b]	*MSH6*	exonic	nonsynonymous SNV	0	0	0	1
2	47806231	C	T	rs63750370	*MSH6*	exonic	nonsynonymous SNV	1	0	0	0
2	58165804	A	G	rs368900762	*FANCL*	exonic	nonsynonymous SNV	0	0	1	0
2	58165817	A	T	rs199660431	*FANCL*	exonic	nonsynonymous SNV	0	0	1	0
2	58241246	G	A	rs750217091	*FANCL*	exonic	nonsynonymous SNV	0	0	0	1
2	99424254	A	C	rs370104816	*REV1*	exonic	nonsynonymous SNV	1	0	0	0
2	99435854	C	T	rs148052685	*REV1*	exonic	nonsynonymous SNV	0	0	0	1
2	99435902	C	T	– [b]	*REV1*	exonic	nonsynonymous SNV	0	0	0	1
2	127271395	T	A	rs753886932	*ERCC3*	exonic	nonsynonymous SNV	0	0	1	0
2	127292767	T	C	rs116713511	*ERCC3*	exonic	nonsynonymous SNV	0	0	1	0
2	127852979	T	C	rs142734461	*POLR2D*	exonic	nonsynonymous SNV	0	0	0	1
2	189795860	C	T	rs61756360	*PMS1*	exonic	nonsynonymous SNV	0	0	0	1
2	189854773	G	A	rs1145232	*PMS1*	exonic	nonsynonymous SNV	12	6	0	0
2	189873575	A	T	– [b]	*PMS1*	exonic	nonsynonymous SNV	0	0	1	0
2	189877284	C	T	rs142159998	*PMS1*	exonic	nonsynonymous SNV	0	0	1	0
2	214730427	A	C	rs556775078	*BARD1*	exonic	nonsynonymous SNV	1	0	0	0
2	214745102	C	T	rs587782252	*BARD1*	exonic	nonsynonymous SNV	0	0	1	0
2	214767497	G	A	rs758139986	*BARD1*	exonic	nonsynonymous SNV	0	0	1	0
2	216860003	C	T	rs148824183	*TNP1*	exonic	nonsynonymous SNV	0	0	0	1
2	219158193	C	T	rs61753339	*NHEJ1*	exonic	nonsynonymous SNV	0	0	0	1
2	231067092	A	C	rs75044156	*PSMD1*	exonic	nonsynonymous SNV	2	2	0	0
2	231166007	C	T	– [b]	*PSMD1*	exonic	nonsynonymous SNV	0	0	1	0

CHROM [a]	POS	REF	ALT	RS_ID	Ref. Gene	Function	Exonic function	African American		European American	
								Lethal cases, No.	Indolent cases, No.	Lethal cases, No.	Indolent cases, No.
2	238275758	C	T	rs150895165	*PER2*	exonic	nonsynonymous SNV	0	3	0	0
3	9750423	G	A	rs104893751	*OGG1*	exonic	nonsynonymous SNV	0	0	2	2
3	9754716	A	G	rs754929964	*OGG1*	exonic	nonsynonymous SNV	0	0	1	0
3	9756791	G	A	rs113561019	*OGG1*	exonic	nonsynonymous SNV	1	0	2	7
3	10049396	C	T	– [b]	*FANCD2*	exonic	nonsynonymous SNV	0	0	0	1
3	10081131	A	T	rs765550790	*FANCD2*	exonic	nonsynonymous SNV	0	0	1	0
3	10085877	G	A	rs755748094	*FANCD2*	exonic	nonsynonymous SNV	0	0	1	0
3	36993599	C	T	rs367654552	*MLH1*	exonic	nonsynonymous SNV	1	0	0	0
3	37004465	G	A	rs730881736	*MLH1*	exonic	nonsynonymous SNV	0	0	0	1
3	37007004	G	C	rs28930073	*MLH1*	exonic	nonsynonymous SNV	0	0	0	1
3	37028794	C	T	rs147939838	*MLH1*	exonic	nonsynonymous SNV	0	0	1	0
3	37028794	C	G	rs147939838	*MLH1*	exonic	nonsynonymous SNV	1	0	0	0
3	37047529	C	T	rs63751684	*MLH1*	exonic	nonsynonymous SNV	1	0	0	0
3	48450162	A	C	rs11925638	*ATRIP*	exonic	nonsynonymous SNV	14	6	0	1
3	51944212	C	G	rs372767757	*PARP3*	exonic	nonsynonymous SNV	0	0	0	1
3	51944495	C	T	rs201155006	*PARP3*	exonic	nonsynonymous SNV	0	0	1	1
3	51945148	C	T	rs79176238	*PARP3*	exonic	nonsynonymous SNV	1	2	0	0
3	51946220	G	A	rs76343500	*PARP3*	exonic	nonsynonymous SNV	19	13	0	1
3	51946265	C	T	rs201317730	*PARP3*	exonic	nonsynonymous SNV	0	1	0	0
3	64010930	C	T	rs140886939	*PSMD6*	exonic	nonsynonymous SNV	1	0	2	0
3	121436275	C	T	rs41540016	*POLQ*	exonic	nonsynonymous SNV	2	1	12	6
3	121449320	T	C	rs150364457	*POLQ*	exonic	nonsynonymous SNV	0	0	1	0
3	121460180	T	C	– [b]	*POLQ*	exonic	nonsynonymous SNV	0	0	0	1
3	121490295	A	G	rs145048812	*POLQ*	exonic	nonsynonymous SNV	0	0	1	0
3	121493650	G	A	rs142004739	*POLQ*	exonic	nonsynonymous SNV	2	0	0	0
3	121496853	G	A	rs373455498	*POLQ*	exonic	nonsynonymous SNV	0	0	0	1

CHROM [a]	POS	REF	ALT	RS_ID	Ref. Gene	Function	Exonic function	African American		European American	
								Lethal cases, No.	Indolent cases, No.	Lethal cases, No.	Indolent cases, No.
3	121496894	T	G	rs138010423	*POLQ*	exonic	nonsynonymous SNV	0	0	1	0
3	129433124	C	T	rs758035705	*MBD4*	exonic	nonsynonymous SNV	0	0	1	0
3	129436658	G	A	– [b]	*MBD4*	exonic	nonsynonymous SNV	0	0	1	0
3	129437734	A	T	rs778846345	*MBD4*	exonic	nonsynonymous SNV	1	0	0	0
3	129437736	C	A	rs201697944	*MBD4*	exonic	nonsynonymous SNV	0	0	1	0
3	131109894	T	C	rs776096000	*NEK11*	exonic	nonsynonymous SNV	1	0	0	0
3	131133881	C	T	rs145728410	*NEK11*	exonic	nonsynonymous SNV	0	0	0	1
3	131162464	C	T	rs61749492	*NEK11*	exonic	nonsynonymous SNV	3	0	0	0
3	133652482	T	A	rs184600255	*TOPBP1*	exonic	nonsynonymous SNV	1	0	0	0
3	142457748	G	T	– [b]	*ATR*	exonic	nonsynonymous SNV	0	0	1	0
3	142459279	G	A	– [b]	*ATR*	exonic	nonsynonymous SNV	0	1	0	0
3	142493187	C	A	rs145569221	*ATR*	exonic	nonsynonymous SNV	0	0	0	1
3	142566172	G	A	rs147353060	*ATR*	exonic	nonsynonymous SNV	0	0	1	0
3	184303660	G	T	rs201414592	*PSMD2*	exonic	nonsynonymous SNV	0	0	1	0
3	186792864	C	T	rs376471242	*RFC4*	exonic	nonsynonymous SNV	0	0	1	0
3	186794727	C	T	rs148403533	*RFC4*	exonic	nonsynonymous SNV	0	1	0	0
4	39300056	G	A	rs752627380	*RFC1*	exonic	nonsynonymous SNV	0	1	0	0
4	56994411	A	G	rs146360510	*POLR2B*	exonic	nonsynonymous SNV	0	0	1	0
4	57005695	C	T	rs199642101	*POLR2B*	exonic	nonsynonymous SNV	0	0	1	0
5	34914838	C	A	rs373008790	*RAD1*	exonic	nonsynonymous SNV	1	0	0	0
5	60898280	G	T	rs61754098	*ERCC8*	exonic	nonsynonymous SNV	0	0	0	1
5	60899662	T	G	rs192183306	*ERCC8*	exonic	nonsynonymous SNV	0	1	0	0
5	60899690	C	G	rs150727525	*ERCC8*	exonic	nonsynonymous SNV	0	0	0	1
5	60918330	T	C	– [b]	*ERCC8*	exonic	nonsynonymous SNV	0	0	0	1
5	69273031	C	T	rs34584424	*CDK7*	exonic	nonsynonymous SNV	0	1	17	14
5	80665215	C	T	– [b]	*MSH3*	exonic	nonsynonymous SNV	1	0	0	0

CHROM [a]	POS	REF	ALT	RS_ID	Ref. Gene	Function	Exonic function	African American		European American	
								Lethal cases, No.	Indolent cases, No.	Lethal cases, No.	Indolent cases, No.
5	80665338	A	G	rs144012714	*MSH3*	exonic	nonsynonymous SNV	1	0	0	0
5	80672813	C	T	rs745412081	*MSH3*	exonic	nonsynonymous SNV	0	0	1	0
5	80678930	G	A	rs761779919	*MSH3*	exonic	nonsynonymous SNV	0	0	1	0
5	80768041	C	T	rs35045151	*MSH3*	exonic	nonsynonymous SNV	1	1	0	0
5	80768840	G	A	– [b]	*MSH3*	exonic	nonsynonymous SNV	1	0	0	0
5	80775702	A	G	rs200819607	*MSH3*	exonic	nonsynonymous SNV	0	0	0	1
5	80813660	T	G	rs41545019	*MSH3*	exonic	nonsynonymous SNV	0	0	1	4
5	80873250	A	C	– [b]	*MSH3*	exonic	nonsynonymous SNV	1	0	0	0
5	83203604	C	T	rs140143447	*XRCC4*	exonic	nonsynonymous SNV	0	1	0	0
5	87401714	C	G	rs753326332	*CCNH*	exonic	nonsynonymous SNV	0	0	0	1
5	87407984	C	T	rs770037305	*CCNH*	exonic	nonsynonymous SNV	0	0	1	0
5	138286053	C	T	rs370578261	*CDC25C*	exonic	nonsynonymous SNV	0	1	0	0
5	138286077	T	G	– [b]	*CDC25C*	exonic	nonsynonymous SNV	0	0	0	1
5	138289539	C	G	rs11567997	*CDC25C*	exonic	nonsynonymous SNV	3	0	22	11
6	2766299	C	T	rs565906761	*WRNIP1*	exonic	nonsynonymous SNV	0	0	0	1
6	2783445	C	T	– [b]	*WRNIP1*	exonic	nonsynonymous SNV	1	0	0	0
6	30714120	G	A	rs755140078	*MDC1*	exonic	nonsynonymous SNV	0	0	0	1
6	30912113	G	A	rs760294963	*GTF2H4*	exonic	nonsynonymous SNV	0	0	0	1
6	31743924	C	T	rs141863919	*MSH5*	exonic	nonsynonymous SNV	0	0	1	0
6	31753315	G	A	rs372287164	*MSH5*	exonic	nonsynonymous SNV	0	0	1	0
6	31758201	C	G	rs28399976	*MSH5*	exonic	nonsynonymous SNV	1	4	7	9
6	31758279	C	T	rs28399977	*MSH5*	exonic	nonsynonymous SNV	3	2	0	0
6	31760212	A	G	rs61748589	*MSH5*	exonic	nonsynonymous SNV	0	0	5	4
6	32842965	C	T	rs780859553	*PSMB8*	exonic	nonsynonymous SNV	0	0	0	1
6	32843017	T	A	rs17220206	*PSMB8*	exonic	nonsynonymous SNV	8	2	2	2
6	33319515	T	G	rs142867298	*DAXX*	exonic	nonsynonymous SNV	0	0	0	1

CHROM [a]	POS	REF	ALT	RS_ID	Ref. Gene	Function	Exonic function	African American		European American	
								Lethal cases, No.	Indolent cases, No.	Lethal cases, No.	Indolent cases, No.
6	35459358	C	T	rs371020401	*FANCE*	exonic	nonsynonymous SNV	0	1	0	0
6	36684301	G	A	rs45548832	*CDKN1A*	exonic	nonsynonymous SNV	1	0	0	0
6	36684451	G	A	rs148679597	*CDKN1A*	exonic	nonsynonymous SNV	0	0	2	2
6	43597839	T	C	rs775225254	*POLH*	exonic	nonsynonymous SNV	1	0	0	0
7	5977638	G	A	rs149202766	*PMS2*	exonic	nonsynonymous SNV	0	1	0	0
7	5977701	A	T	– [b]	*PMS2*	exonic	nonsynonymous SNV	0	0	1	0
7	5982849	C	T	rs201671325	*PMS2*	exonic	nonsynonymous SNV	0	0	2	2
7	5999241	T	C	rs375289386	*PMS2*	exonic	nonsynonymous SNV	0	1	0	0
7	6002493	A	G	rs116349687	*PMS2*	exonic	nonsynonymous SNV	2	0	0	0
7	6002515	C	T	rs142416537	*PMS2*	exonic	nonsynonymous SNV	0	0	0	1
7	6005969	C	G	rs146176004	*PMS2*	exonic	nonsynonymous SNV	0	0	1	0
7	6006002	A	G	rs201343342	*PMS2*	exonic	nonsynonymous SNV	0	0	0	1
7	7637073	A	T	– [b]	*RPA3*	exonic	nonsynonymous SNV	0	1	0	0
7	44076611	C	T	rs142658845	*POLM*	exonic	nonsynonymous SNV	0	0	1	0
7	44078795	C	G	rs28382644	*POLM*	exonic	nonsynonymous SNV	0	0	5	7
7	44082284	C	T	rs757100319	*POLM*	exonic	nonsynonymous SNV	0	0	0	1
7	74239990	G	A	– [b]	*RFC2*	exonic	nonsynonymous SNV	0	0	1	0
7	105565375	T	C	rs139459919	*RINT1*	exonic	nonsynonymous SNV	0	0	0	2
7	152648677	A	C	rs145085742	*XRCC2*	exonic	nonsynonymous SNV	4	0	0	0
7	152649291	C	T	– [b]	*XRCC2*	exonic	nonsynonymous SNV	0	0	0	1
8	9720477	C	T	rs755395371	*TNKS*	exonic	nonsynonymous SNV	1	0	0	0
8	9735409	C	G	– [b]	*TNKS*	exonic	nonsynonymous SNV	0	0	0	1
8	47912537	C	T	– [b]	*PRKDC*	exonic	nonsynonymous SNV	0	0	1	0
8	47935841	G	T	rs61729514	*PRKDC*	exonic	nonsynonymous SNV	2	1	0	0
8	89937033	G	T	– [b]	*NBN*	exonic	nonsynonymous SNV	1	0	0	0
8	89970472	A	G	rs147626427	*NBN*	exonic	nonsynonymous SNV	2	0	0	0

CHROM [a]	POS	REF	ALT	RS_ID	Ref. Gene	Function	Exonic function	African American		European American	
								Lethal cases, No.	Indolent cases, No.	Lethal cases, No.	Indolent cases, No.
8	89971222	C	A	rs786202250	*NBN*	exonic	nonsynonymous SNV	1	0	0	0
8	89971232	G	A	rs34767364	*NBN*	exonic	nonsynonymous SNV	1	0	2	1
8	89980853	C	A	– [b]	*NBN*	exonic	nonsynonymous SNV	0	1	0	0
8	89981412	C	T	rs61753720	*NBN*	exonic	nonsynonymous SNV	0	0	1	4
8	94387080	T	A	rs144828525	*RAD54B*	exonic	nonsynonymous SNV	0	0	0	1
8	116863204	T	C	– [b]	*RAD21*	exonic	nonsynonymous SNV	0	1	0	0
9	32989874	C	A	rs144076460	*APTX*	exonic	nonsynonymous SNV	0	0	0	1
9	35061163	T	G	– [b]	*VCP*	exonic	nonsynonymous SNV	0	0	0	1
9	95150025	T	C	– [b]	*FANCC*	exonic	nonsynonymous SNV	0	0	0	1
9	97675521	C	T	rs749724416	*XPA*	exonic	nonsynonymous SNV	0	1	0	0
9	97689601	A	G	– [b]	*XPA*	exonic	nonsynonymous SNV	0	0	1	0
9	132288565	C	G	rs755531730	*SETX*	exonic	nonsynonymous SNV	0	0	0	1
9	132288636	A	G	rs140676924	*SETX*	exonic	nonsynonymous SNV	0	0	0	1
9	132295965	C	T	rs148568105	*SETX*	exonic	nonsynonymous SNV	0	1	0	0
9	132326938	A	C	rs112089123	*SETX*	exonic	nonsynonymous SNV	1	0	3	0
9	132327078	T	G	rs138195434	*SETX*	exonic	nonsynonymous SNV	1	0	0	0
9	132327775	G	A	rs760272692	*SETX*	exonic	nonsynonymous SNV	1	0	0	0
9	132327783	T	C	rs202204341	*SETX*	exonic	nonsynonymous SNV	0	0	1	0
9	132327935	C	G	rs12344006	*SETX*	exonic	nonsynonymous SNV	4	0	0	0
9	132329197	T	C	rs149718424	*SETX*	exonic	nonsynonymous SNV	1	0	0	0
10	14926856	G	C	rs41298896	*DCLRE1C*	exonic	nonsynonymous SNV	8	1	0	0
10	14934465	G	A	rs772391197	*DCLRE1C*	exonic	nonsynonymous SNV	1	0	0	0
10	14945139	G	A	rs147013097	*DCLRE1C*	exonic	nonsynonymous SNV	0	0	1	1
10	14945182	C	A	rs138077101	*DCLRE1C*	exonic	nonsynonymous SNV	0	0	0	1
10	49459086	C	T	rs755854972	*ERCC6*	exonic	nonsynonymous SNV	0	1	0	0
10	49470310	A	C	rs61760166	*ERCC6*	exonic	nonsynonymous SNV	0	0	1	1

CHROM [a]	POS	REF	ALT	RS_ID	Ref. Gene	Function	Exonic function	African American		European American	
								Lethal cases, No.	Indolent cases, No.	Lethal cases, No.	Indolent cases, No.
10	49470975	C	G	– [b]	*ERCC6*	exonic	nonsynonymous SNV	0	1	0	0
10	49470984	T	C	rs41562713	*ERCC6*	exonic	nonsynonymous SNV	0	0	1	0
10	49471041	A	T	rs747581337	*ERCC6*	exonic	nonsynonymous SNV	1	0	0	0
10	49471055	T	G	rs766459533	*ERCC6*	exonic	nonsynonymous SNV	0	0	1	0
10	49472376	C	T	rs145720191	*ERCC6*	exonic	nonsynonymous SNV	0	0	2	1
10	49472994	C	T	rs753804966	*ERCC6*	exonic	nonsynonymous SNV	0	0	0	1
10	49473574	A	G	– [b]	*ERCC6*	exonic	nonsynonymous SNV	0	0	0	1
10	49476298	G	A	rs370105701	*ERCC6*	exonic	nonsynonymous SNV	0	0	0	1
10	49482760	G	A	rs55698015	*ERCC6*	exonic	nonsynonymous SNV	1	0	0	0
10	49482775	G	A	rs114852424	*ERCC6*	exonic	nonsynonymous SNV	1	0	0	0
10	49482779	A	T	rs199921831	*ERCC6*	exonic	nonsynonymous SNV	0	0	0	1
10	49482860	G	A	rs61760163	*ERCC6*	exonic	nonsynonymous SNV	0	0	2	1
10	49524156	T	G	rs4253046	*ERCC6*	exonic	nonsynonymous SNV	0	0	2	0
10	49530733	T	C	rs141372606	*ERCC6*	exonic	nonsynonymous SNV	0	0	1	0
10	72161556	C	A	– [b]	*ASCC1*	exonic	nonsynonymous SNV	0	0	1	0
10	96304639	C	A	rs779136442	*DNTT*	exonic	nonsynonymous SNV	0	0	1	0
10	96332529	G	A	rs142389547	*DNTT*	exonic	nonsynonymous SNV	0	0	1	0
10	97459234	G	A	rs200490757	*MMS19*	exonic	nonsynonymous SNV	0	0	1	2
10	97460142	G	C	rs191066247	*MMS19*	exonic	nonsynonymous SNV	0	1	0	0
10	97460705	A	C	rs374461373	*MMS19*	exonic	nonsynonymous SNV	1	0	0	0
10	101582831	C	T	rs200623399	*POLL*	exonic	nonsynonymous SNV	0	0	0	1
10	101582891	C	T	rs139871590	*POLL*	exonic	nonsynonymous SNV	0	1	0	0
10	101583557	G	A	rs146197224	*POLL*	exonic	nonsynonymous SNV	0	0	0	1
10	101583666	G	A	rs142726673	*POLL*	exonic	nonsynonymous SNV	0	0	2	1
10	101586103	G	A	rs61757734	*POLL*	exonic	nonsynonymous SNV	0	0	1	0
10	113842433	T	A	rs11196530	*DCLRE1A*	exonic	nonsynonymous SNV	0	0	2	4

CHROM [a]	POS	REF	ALT	RS_ID	Ref. Gene	Function	Exonic function	African American		European American	
								Lethal cases, No.	Indolent cases, No.	Lethal cases, No.	Indolent cases, No.
10	113847315	C	T	rs765337471	*DCLRE1A*	exonic	nonsynonymous SNV	0	0	0	1
10	113849394	C	G	rs777776912	*DCLRE1A*	exonic	nonsynonymous SNV	0	0	1	0
11	840433	A	C	– [b]	*POLR2L*	exonic	nonsynonymous SNV	1	0	0	0
11	45858827	G	A	– [b]	*CRY2*	exonic	nonsynonymous SNV	0	0	1	0
11	45870427	G	T	– [b]	*CRY2*	exonic	nonsynonymous SNV	0	1	0	0
11	47216874	T	G	rs144729572	*DDB2*	exonic	nonsynonymous SNV	0	0	1	0
11	61312079	G	T	– [b]	*DDB1*	exonic	nonsynonymous SNV	1	0	0	0
11	61314321	G	A	rs777664894	*DDB1*	exonic	nonsynonymous SNV	1	0	0	0
11	61795501	G	A	rs140458864	*FEN1*	exonic	nonsynonymous SNV	0	0	0	1
11	67352749	G	T	rs201016876	*POLD4*	exonic	nonsynonymous SNV	0	0	1	0
11	67393610	G	A	rs761540694	*RAD9A*	exonic	nonsynonymous SNV	0	0	1	0
11	68904010	G	A	rs774019567	*IGHMBP2*	exonic	nonsynonymous SNV	0	0	1	0
11	68908250	C	T	rs2228205	*IGHMBP2*	exonic	nonsynonymous SNV	0	1	0	0
11	68908535	G	A	rs536962342	*IGHMBP2*	exonic	nonsynonymous SNV	0	0	1	0
11	68908539	T	C	– [b]	*IGHMBP2*	exonic	nonsynonymous SNV	0	0	0	1
11	68914878	C	G	rs148095551	*IGHMBP2*	exonic	nonsynonymous SNV	0	0	0	1
11	68929186	C	T	rs142062146	*IGHMBP2*	exonic	nonsynonymous SNV	1	0	0	0
11	68929315	C	T	rs35193202	*IGHMBP2*	exonic	nonsynonymous SNV	0	0	0	1
11	68939547	G	A	– [b]	*IGHMBP2*	exonic	nonsynonymous SNV	0	0	1	0
11	68939571	G	A	rs373408947	*IGHMBP2*	exonic	nonsynonymous SNV	0	0	1	0
11	68939671	T	G	rs147674615	*IGHMBP2*	exonic	nonsynonymous SNV	0	0	0	3
11	94464175	C	T	rs587780134	*MRE11*	exonic	nonsynonymous SNV	1	0	0	0
11	94471743	T	C	– [b]	*MRE11*	exonic	nonsynonymous SNV	0	0	0	1
11	94479718	T	C	rs372131911	*MRE11*	exonic	nonsynonymous SNV	0	0	1	0
11	108229287	A	G	rs137882485	*ATM*	exonic	nonsynonymous SNV	0	0	0	2
11	108235672	G	A	rs146382972	*ATM*	exonic	nonsynonymous SNV	1	0	0	0

CHROM [a]	POS	REF	ALT	RS_ID	Ref. Gene	Function	Exonic function	African American		European American	
								Lethal cases, No.	Indolent cases, No.	Lethal cases, No.	Indolent cases, No.
11	108254011	A	G	rs147934285	*ATM*	exonic	nonsynonymous SNV	1	0	0	0
11	108271086	T	G	– [b]	*ATM*	exonic	nonsynonymous SNV	1	0	0	0
11	108281097	G	A	rs200765255	*ATM*	exonic	nonsynonymous SNV	0	0	1	0
11	108289740	G	A	rs145667735	*ATM*	exonic	nonsynonymous SNV	0	0	1	0
11	108289753	T	G	rs138327406	*ATM*	exonic	nonsynonymous SNV	0	0	0	6
11	108292687	G	T	rs759340881	*ATM*	exonic	nonsynonymous SNV	0	0	0	1
11	108293425	G	A	rs550552791	*ATM*	exonic	nonsynonymous SNV	0	0	1	0
11	108301655	G	C	rs3092907	*ATM*	exonic	nonsynonymous SNV	0	0	0	1
11	108307950	C	A	rs143577586	*ATM*	exonic	nonsynonymous SNV	0	0	1	0
11	108321391	G	T	rs138828590	*ATM*	exonic	nonsynonymous SNV	1	0	0	0
11	108326070	G	A	rs567060474	*ATM*	exonic	nonsynonymous SNV	0	0	1	0
11	108326169	C	T	rs56009889	*ATM*	exonic	nonsynonymous SNV	0	0	2	2
11	108343280	T	C	rs746475628	*ATM*	exonic	nonsynonymous SNV	0	0	1	0
11	108365491	T	C	– [b]	*ATM*	exonic	nonsynonymous SNV	0	0	1	0
11	119095173	G	C	rs780380122	*H2AFX*	exonic	nonsynonymous SNV	1	0	0	0
12	21475555	C	T	rs375822444	*RECQL*	exonic	nonsynonymous SNV	0	0	0	1
12	21486574	C	T	rs138278747	*RECQL*	exonic	nonsynonymous SNV	1	0	0	0
12	54182389	C	T	rs752448480	*SMUG1*	exonic	nonsynonymous SNV	0	0	1	0
12	54182596	G	A	rs3136389	*SMUG1*	exonic	nonsynonymous SNV	1	0	1	0
12	54183729	C	T	rs549646851	*SMUG1*	exonic	nonsynonymous SNV	0	0	0	1
12	56431588	G	T	rs758113731	*TIMELESS*	exonic	nonsynonymous SNV	0	1	0	0
12	102124025	C	T	rs768649483	*PARPBP*	exonic	nonsynonymous SNV	0	0	0	1
12	102148381	G	A	rs80135324	*PARPBP*	exonic	nonsynonymous SNV	5	1	0	0
12	109097711	T	C	rs747684225	*UNG*	exonic	nonsynonymous SNV	0	0	0	1
12	109098561	C	T	rs151095402	*UNG*	exonic	nonsynonymous SNV	0	0	2	1
12	109101993	C	T	– [b]	*UNG*	exonic	nonsynonymous SNV	0	0	1	0

CHROM [a]	POS	REF	ALT	RS_ID	Ref. Gene	Function	Exonic function	African American		European American	
								Lethal cases, No.	Indolent cases, No.	Lethal cases, No.	Indolent cases, No.
12	118026913	G	A	rs748990326	*RFC5*	exonic	nonsynonymous SNV	0	0	0	1
12	123659582	C	G	rs149957130	*GTF2H3*	exonic	nonsynonymous SNV	1	0	0	0
12	132626140	A	G	rs138094751	*POLE*	exonic	nonsynonymous SNV	0	0	0	1
12	132672691	C	T	– [b]	*POLE*	exonic	nonsynonymous SNV	0	0	0	1
12	132675486	C	T	rs199746481	*POLE*	exonic	nonsynonymous SNV	0	0	0	1
12	132676107	T	C	rs5744760	*POLE*	exonic	nonsynonymous SNV	8	5	0	0
12	132677624	T	C	– [b]	*POLE*	exonic	nonsynonymous SNV	0	1	0	0
13	24442591	C	T	rs142527575	*PARP4*	exonic	nonsynonymous SNV	1	1	0	0
13	24442656	A	C	– [b]	*PARP4*	exonic	nonsynonymous SNV	1	0	0	0
13	24469079	T	C	– [b]	*PARP4*	exonic	nonsynonymous SNV	1	0	0	0
13	24503664	G	A	rs141289254	*PARP4*	exonic	nonsynonymous SNV	1	2	4	4
13	32338940	G	A	rs28897728	*BRCA2*	exonic	nonsynonymous SNV	0	0	1	0
13	32341158	G	A	rs80358906	*BRCA2*	exonic	nonsynonymous SNV	0	1	0	0
13	32344569	A	G	rs56272235	*BRCA2*	exonic	nonsynonymous SNV	0	0	1	0
13	32379344	G	T	rs587781762	*BRCA2*	exonic	nonsynonymous SNV	0	0	0	1
13	33821213	C	T	rs372320843	*RFC3*	exonic	nonsynonymous SNV	0	0	0	1
13	33825809	G	A	rs146557081	*RFC3*	exonic	nonsynonymous SNV	0	1	0	0
13	33836185	C	T	rs143372384	*RFC3*	exonic	nonsynonymous SNV	0	0	2	0
13	102868136	A	C	rs752326263	*ERCC5*	exonic	nonsynonymous SNV	1	0	0	0
13	102872337	G	A	rs146344855	*ERCC5*	exonic	nonsynonymous SNV	0	0	1	0
13	102872396	A	C	rs41281674	*ERCC5*	exonic	nonsynonymous SNV	2	0	0	0
13	108209665	G	A	rs374074342	*LIG4*	exonic	nonsynonymous SNV	1	0	0	0
13	108209756	G	A	rs375284799	*LIG4*	exonic	nonsynonymous SNV	1	0	0	0
13	108210289	A	G	– [b]	*LIG4*	exonic	nonsynonymous SNV	0	0	0	1
13	108210446	C	G	– [b]	*LIG4*	exonic	nonsynonymous SNV	0	0	1	0
13	108210460	A	G	– [b]	*LIG4*	exonic	nonsynonymous SNV	1	0	0	0

CHROM [a]	POS	REF	ALT	RS_ID	Ref. Gene	Function	Exonic function	African American		European American	
								Lethal cases, No.	Indolent cases, No.	Lethal cases, No.	Indolent cases, No.
13	113254968	G	A	rs771850019	*CUL4A*	exonic	nonsynonymous SNV	0	0	1	0
13	113254985	C	G	rs762083792	*CUL4A*	exonic	nonsynonymous SNV	0	0	0	1
13	113263557	C	T	– [b]	*CUL4A*	exonic	nonsynonymous SNV	0	1	0	0
14	20356663	C	A	rs748252703	*PARP2*	exonic	nonsynonymous SNV	0	0	0	1
14	20455917	C	T	rs150934075	*APEX1*	exonic	nonsynonymous SNV	0	0	0	1
14	20455958	A	C	rs61757709	*APEX1*	exonic	nonsynonymous SNV	0	0	1	0
14	20457105	G	C	rs750459021	*APEX1*	exonic	nonsynonymous SNV	0	0	0	1
14	20457272	G	A	rs33956927	*APEX1*	exonic	nonsynonymous SNV	5	3	0	0
14	34765226	C	T	rs780846512	*BAZ1A*	exonic	nonsynonymous SNV	0	0	1	0
14	34776297	A	C	– [b]	*BAZ1A*	exonic	nonsynonymous SNV	0	1	0	0
14	45164374	C	T	rs146151355	*FANCM*	exonic	nonsynonymous SNV	0	0	1	1
14	45175750	C	T	rs148304968	*FANCM*	exonic	nonsynonymous SNV	1	0	0	0
14	52707211	C	T	rs371829618	*PSMC6*	UTR5	– [b]	0	0	0	1
14	60808335	G	C	rs148083759	*MNAT1*	exonic	nonsynonymous SNV	2	0	0	0
14	75017093	C	T	rs28939071	*MLH3*	exonic	nonsynonymous SNV	0	0	0	2
14	75033445	C	T	rs781739661	*MLH3*	exonic	nonsynonymous SNV	0	0	0	1
14	75039943	G	A	rs761024324	*MLH3*	exonic	nonsynonymous SNV	0	1	0	0
14	75039953	C	G	– [b]	*MLH3*	exonic	nonsynonymous SNV	1	0	0	0
14	75041640	T	A	rs142124529	*MLH3*	exonic	nonsynonymous SNV	0	0	1	0
14	75046439	C	T	rs28756993	*MLH3*	exonic	nonsynonymous SNV	4	2	1	0
14	75048422	T	C	rs61754769	*MLH3*	exonic	nonsynonymous SNV	0	0	1	1
14	75048742	A	G	rs150796590	*MLH3*	exonic	nonsynonymous SNV	0	1	0	0
14	75048754	T	A	rs77687901	*MLH3*	exonic	nonsynonymous SNV	0	1	0	0
14	75049297	A	G	rs28756979	*MLH3*	exonic	nonsynonymous SNV	1	0	0	0
14	89162461	G	A	rs370136184	*FOXN3*	exonic	nonsynonymous SNV	0	0	0	1
14	89180708	C	G	rs74076959	*FOXN3*	exonic	nonsynonymous SNV	1	0	0	1

CHROM [a]	POS	REF	ALT	RS_ID	Ref. Gene	Function	Exonic function	African American		European American	
								Lethal cases, No.	Indolent cases, No.	Lethal cases, No.	Indolent cases, No.
14	89989742	G	A	rs144746398	*TDP1*	exonic	nonsynonymous SNV	0	0	1	1
14	103699479	C	A	– [b]	*XRCC3*	exonic	nonsynonymous SNV	0	0	1	0
14	103707018	C	T	rs750840706	*XRCC3*	exonic	nonsynonymous SNV	0	1	0	0
14	103707123	C	T	– [b]	*XRCC3*	exonic	nonsynonymous SNV	0	0	1	0
15	40701137	A	G	rs769146109	*RAD51*	exonic	nonsynonymous SNV	0	0	1	0
15	40718821	G	C	– [b]	*RAD51*	exonic	nonsynonymous SNV	1	0	0	0
15	40718842	T	C	– [b]	*RAD51*	exonic	nonsynonymous SNV	0	0	0	1
15	40728713	A	G	rs779214128	*RAD51*	exonic	nonsynonymous SNV	1	0	0	0
15	43447402	G	C	rs748173675	*TP53BP1*	exonic	nonsynonymous SNV	1	0	0	0
15	43447470	A	C	– [b]	*TP53BP1*	exonic	nonsynonymous SNV	0	0	1	0
15	75349153	G	A	rs5745906	*NEIL1*	exonic	nonsynonymous SNV	0	0	0	3
15	75352173	T	C	– [b]	*NEIL1*	exonic	nonsynonymous SNV	0	0	1	0
15	75352179	A	C	– [b]	*NEIL1*	exonic	nonsynonymous SNV	0	0	0	1
15	75353853	C	T	rs142213781	*NEIL1*	exonic	nonsynonymous SNV	0	0	1	0
15	89281825	A	G	rs144908351	*FANCI*	exonic	nonsynonymous SNV	0	0	1	0
15	89305390	C	T	rs191202700	*FANCI*	exonic	nonsynonymous SNV	1	0	0	0
15	89316791	G	T	rs775517153	*POLG*	exonic	nonsynonymous SNV	0	0	0	1
15	89317460	G	A	rs369544574	*POLG*	exonic	nonsynonymous SNV	0	0	0	1
15	89319028	T	C	rs201192905	*POLG*	exonic	nonsynonymous SNV	0	0	1	0
15	89319034	A	G	– [b]	*POLG*	exonic	nonsynonymous SNV	0	0	1	0
15	89321768	C	T	– [b]	*POLG*	exonic	nonsynonymous SNV	0	1	0	0
15	89321780	G	A	rs144500145	*POLG*	exonic	nonsynonymous SNV	0	0	0	1
15	89323423	A	G	rs202037973	*POLG*	exonic	nonsynonymous SNV	1	0	0	0
15	89323426	C	G	rs113994097	*POLG*	exonic	nonsynonymous SNV	0	0	0	1
15	89323460	C	G	rs121918054	*POLG*	exonic	nonsynonymous SNV	0	0	2	1
15	89326688	G	A	rs2307447	*POLG*	exonic	nonsynonymous SNV	1	2	0	0

CHROM [a]	POS	REF	ALT	RS_ID	Ref. Gene	Function	Exonic function	African American		European American	
								Lethal cases, No.	Indolent cases, No.	Lethal cases, No.	Indolent cases, No.
15	89327315	C	G	– [b]	*POLG*	exonic	nonsynonymous SNV	0	1	0	0
15	89328532	G	C	rs145289229	*POLG*	exonic	nonsynonymous SNV	0	0	2	0
15	89330133	C	G	rs61752784	*POLG*	exonic	nonsynonymous SNV	0	0	2	3
15	89330197	G	C	rs754696832	*POLG*	exonic	nonsynonymous SNV	0	0	0	1
15	89333367	G	A	rs201261842	*POLG*	exonic	nonsynonymous SNV	0	0	1	0
15	90763047	T	C	rs748567176	*BLM*	exonic	nonsynonymous SNV	0	0	1	0
15	90785002	C	T	rs775026151	*BLM*	exonic	nonsynonymous SNV	1	0	0	0
16	2044652	A	G	rs1805378	*NTHL1*	exonic	nonsynonymous SNV	0	0	1	5
16	2044700	G	A	rs756403102	*NTHL1*	exonic	nonsynonymous SNV	0	0	1	0
16	2046208	G	A	rs148104494	*NTHL1*	exonic	nonsynonymous SNV	0	0	2	1
16	2046240	G	A	– [b]	*NTHL1*	exonic	nonsynonymous SNV	0	0	0	1
16	13934224	C	T	rs1799802	*ERCC4*	exonic	nonsynonymous SNV	0	1	5	4
16	13935695	T	C	rs371392134	*ERCC4*	exonic	nonsynonymous SNV	0	1	0	0
16	13944827	G	A	rs56129764	*ERCC4*	exonic	nonsynonymous SNV	0	0	0	1
16	13947661	C	A	rs149364215	*ERCC4*	exonic	nonsynonymous SNV	0	0	1	0
16	13948096	G	T	rs138583819	*ERCC4*	exonic	nonsynonymous SNV	0	0	0	1
16	57462749	G	A	– [b]	*POLR2C*	exonic	nonsynonymous SNV	1	0	0	0
16	57466018	C	T	rs199769391	*POLR2C*	exonic	nonsynonymous SNV	0	0	0	1
16	57471010	G	A	rs150877837	*POLR2C*	exonic	nonsynonymous SNV	0	0	0	1
16	89745061	G	A	rs147017625	*FANCA*	exonic	nonsynonymous SNV	0	1	0	0
16	89746667	G	A	rs143671872	*FANCA*	exonic	nonsynonymous SNV	0	0	0	1
16	89746848	T	C	rs574034197	*FANCA*	exonic	nonsynonymous SNV	0	0	1	0
16	89770606	C	T	– [b]	*FANCA*	exonic	nonsynonymous SNV	0	0	1	0
16	89775768	C	G	rs139235751	*FANCA*	exonic	nonsynonymous SNV	0	0	3	1
16	89778969	G	C	rs145459403	*FANCA*	exonic	nonsynonymous SNV	0	0	1	0
16	89779908	T	C	rs753229112	*FANCA*	exonic	nonsynonymous SNV	0	0	1	0

CHROM [a]	POS	REF	ALT	RS_ID	Ref. Gene	Function	Exonic function	African American		European American	
								Lethal cases, No.	Indolent cases, No.	Lethal cases, No.	Indolent cases, No.
16	89810724	C	G	rs372691338	*FANCA*	exonic	nonsynonymous SNV	1	0	0	0
17	1883879	G	A	rs142406311	*RPA1*	exonic	nonsynonymous SNV	1	0	0	0
17	7674876	G	A	– [b]	*TP53*	exonic	nonsynonymous SNV	0	0	0	1
17	7674944	C	T	– [b]	*TP53*	exonic	nonsynonymous SNV	0	0	0	1
17	7675085	C	T	rs786202962	*TP53*	exonic	nonsynonymous SNV	1	0	0	0
17	7675145	C	T	rs371524413	*TP53*	exonic	nonsynonymous SNV	0	0	0	1
17	18278013	T	C	rs538053478	*TOP3A*	exonic	nonsynonymous SNV	1	0	0	0
17	18292713	G	A	rs76300532	*TOP3A*	exonic	nonsynonymous SNV	12	9	0	0
17	18302619	C	T	rs117856165	*TOP3A*	exonic	nonsynonymous SNV	0	0	1	1
17	35002755	G	A	rs149236110	*LIG3*	exonic	nonsynonymous SNV	0	0	1	0
17	35103501	G	A	rs370228071	*RAD51D*	exonic	nonsynonymous SNV	0	0	1	0
17	35107046	A	G	rs780938875	*RAD51D*	exonic	nonsynonymous SNV	0	0	0	1
17	35107074	C	T	rs201141245	*RAD51D*	exonic	nonsynonymous SNV	0	0	1	0
17	35107118	C	A	– [b]	*RAD51D*	exonic	nonsynonymous SNV	1	0	0	0
17	39984325	A	C	– [b]	*PSMD3*	exonic	nonsynonymous SNV	0	0	0	1
17	39984468	T	C	– [b]	*PSMD3*	exonic	nonsynonymous SNV	0	1	0	0
17	39988780	G	A	rs139137910	*PSMD3*	exonic	nonsynonymous SNV	0	0	1	0
17	43049179	A	G	rs55808233	*BRCA1*	exonic	nonsynonymous SNV	0	1	0	0
17	43057077	C	T	rs80357442	*BRCA1*	exonic	nonsynonymous SNV	1	0	0	0
17	43057122	A	G	rs45553935	*BRCA1*	exonic	nonsynonymous SNV	0	0	1	0
17	43071232	G	A	rs56158747	*BRCA1*	exonic	nonsynonymous SNV	1	2	0	0
17	43074471	C	A	rs1800744	*BRCA1*	exonic	nonsynonymous SNV	0	0	6	0
17	43091002	G	C	rs80356986	*BRCA1*	exonic	nonsynonymous SNV	0	0	1	0
17	43092451	C	T	rs80357386	*BRCA1*	exonic	nonsynonymous SNV	0	0	1	0
17	43092706	G	T	– [b]	*BRCA1*	exonic	nonsynonymous SNV	0	1	0	0
17	43092935	G	A	rs41286300	*BRCA1*	exonic	nonsynonymous SNV	0	1	0	1

CHROM [a]	POS	REF	ALT	RS_ID	Ref. Gene	Function	Exonic function	African American		European American	
								Lethal cases, No.	Indolent cases, No.	Lethal cases, No.	Indolent cases, No.
17	43093309	G	A	rs80357051	*BRCA1*	exonic	nonsynonymous SNV	0	0	1	0
17	43093883	T	G	rs56012641	*BRCA1*	exonic	nonsynonymous SNV	0	0	1	0
17	43093997	G	A	rs41286294	*BRCA1*	exonic	nonsynonymous SNV	0	0	1	0
17	43099786	T	C	rs56187033	*BRCA1*	exonic	nonsynonymous SNV	0	0	1	0
17	43104249	T	C	rs28897673	*BRCA1*	exonic	nonsynonymous SNV	1	0	1	0
17	43124066	C	G	– [b]	*BRCA1*	exonic	nonsynonymous SNV	1	0	0	0
17	58703280	T	C	rs201529791	*RAD51C*	exonic	nonsynonymous SNV	0	0	0	1
17	58720761	C	G	– [b]	*RAD51C*	exonic	nonsynonymous SNV	0	0	0	1
17	61784309	A	G	– [b]	*BRIP1*	exonic	nonsynonymous SNV	0	0	0	1
17	61808531	T	C	rs141055990	*BRIP1*	exonic	nonsynonymous SNV	1	0	0	0
17	61847211	G	A	rs4988345	*BRIP1*	exonic	nonsynonymous SNV	0	1	1	4
17	63829943	G	C	– [b]	*PSMC5*	exonic	nonsynonymous SNV	0	0	0	1
17	75627670	C	T	rs200535477	*RECQL5*	exonic	nonsynonymous SNV	0	0	3	1
17	75629133	T	A	rs201921330	*RECQL5*	exonic	nonsynonymous SNV	0	0	0	1
17	75631637	C	T	rs200877940	*RECQL5*	exonic	nonsynonymous SNV	0	0	2	1
17	75666476	A	G	– [b]	*RECQL5*	exonic	nonsynonymous SNV	1	0	0	0
18	23016899	G	T	– [b]	*RBBP8*	exonic	nonsynonymous SNV	1	0	0	0
18	23022190	G	A	rs140196819	*RBBP8*	exonic	nonsynonymous SNV	0	0	0	1
18	54271468	T	C	rs754176652	*POLI*	exonic	nonsynonymous SNV	0	0	1	0
18	54293839	T	C	rs3218786	*POLI*	exonic	nonsynonymous SNV	2	1	13	19
19	7623781	C	T	rs370229927	*XAB2*	exonic	nonsynonymous SNV	0	1	0	0
19	7624361	C	T	rs61761630	*XAB2*	exonic	nonsynonymous SNV	0	0	3	1
19	7624436	C	G	– [b]	*XAB2*	exonic	nonsynonymous SNV	0	0	1	0
19	7627353	G	A	rs745761079	*XAB2*	exonic	nonsynonymous SNV	0	0	1	0
19	43552143	A	T	– [b]	*XRCC1*	exonic	nonsynonymous SNV	0	0	1	0
19	45351681	A	G	rs201370106	*ERCC2*	exonic	nonsynonymous SNV	0	0	1	0

CHROM [a]	POS	REF	ALT	RS_ID	Ref. Gene	Function	Exonic function	African American		European American	
								Lethal cases, No.	Indolent cases, No.	Lethal cases, No.	Indolent cases, No.
19	45352316	G	A	rs201392911	*ERCC2*	exonic	nonsynonymous SNV	0	0	0	1
19	45352648	G	A	rs34517175	*ERCC2*	exonic	nonsynonymous SNV	0	0	2	1
19	45352761	C	G	rs200665173	*ERCC2*	exonic	nonsynonymous SNV	0	0	1	0
19	45353139	C	T	rs147224585	*ERCC2*	exonic	nonsynonymous SNV	0	0	1	0
19	45354762	T	C	– [b]	*ERCC2*	exonic	nonsynonymous SNV	0	0	1	0
19	45354789	C	T	rs142568756	*ERCC2*	exonic	nonsynonymous SNV	0	0	1	0
19	45357289	C	T	rs150865508	*ERCC2*	exonic	nonsynonymous SNV	0	0	0	1
19	45357630	C	T	– [b]	*ERCC2*	exonic	nonsynonymous SNV	0	0	1	0
19	45365146	G	A	rs372425466	*ERCC2*	exonic	nonsynonymous SNV	0	0	0	2
19	48117686	C	A	rs145821638	*LIG1*	exonic	nonsynonymous SNV	0	0	0	1
19	48123308	C	T	rs200049505	*LIG1*	exonic	nonsynonymous SNV	0	0	1	0
19	48123309	G	A	rs55950593	*LIG1*	exonic	nonsynonymous SNV	0	0	0	1
19	49862432	G	A	rs372148913	*PNKP*	exonic	nonsynonymous SNV	0	0	0	1
19	49862444	A	G	– [b]	*PNKP*	exonic	nonsynonymous SNV	0	0	0	1
19	49862581	G	A	rs145615734	*PNKP*	exonic	nonsynonymous SNV	1	0	0	0
19	49863723	G	A	rs755468325	*PNKP*	exonic	nonsynonymous SNV	0	0	1	0
19	49864037	C	T	rs199705876	*PNKP*	exonic	nonsynonymous SNV	0	1	0	0
19	50415779	G	A	rs746950229	*POLD1*	exonic	nonsynonymous SNV	0	0	1	0
19	53889907	G	A	rs770016586	*PRKCG*	exonic	nonsynonymous SNV	0	0	1	0
19	55306438	A	G	rs761823157	*BRSK1*	exonic	nonsynonymous SNV	0	0	0	1
20	1163147	C	T	rs147039294	*PSMF1*	exonic	nonsynonymous SNV	0	1	1	1
20	1164437	G	A	rs148156083	*PSMF1*	exonic	nonsynonymous SNV	0	0	1	0

[a] Chromosome and position based on human genome build 38 (GRCh38).
[b] Not available/applicable
